# A putative WAVE regulatory complex (WRC) interacting receptor sequence (WIRS) in the cytoplasmic tail of HSV-1 gE does not function in WRC recruitment or neuronal transport

**DOI:** 10.1099/acmi.0.000206

**Published:** 2021-03-04

**Authors:** Christopher E. Denes, Timothy P. Newsome, Monica Miranda-Saksena, Anthony L. Cunningham, Russell J. Diefenbach

**Affiliations:** ^1^​ Centre for Virus Research, The Westmead Institute for Medical Research, The University of Sydney, Westmead, NSW 2145, Australia; ^2^​ School of Medical Sciences, Faculty of Medicine and Health, The University of Sydney, Sydney, NSW 2006, Australia; ^3^​ School of Life and Environmental Sciences, Faculty of Science, The University of Sydney, Sydney, NSW 2006, Australia; ^4^​ Department of Biomedical Sciences, Faculty of Medicine, Health and Human Sciences, Macquarie University, Sydney, NSW 2109, Australia

**Keywords:** herpes simplex virus, gE, egress, Arp2/3 complex, WAVE Regulatory Complex, WRC Interacting Receptor Sequence

## Abstract

HSV-1 envelope glycoprotein E (gE) is important for viral egress and cell-to-cell spread but the host protein(s) involved in these functions have yet to be determined. We aimed to investigate a role for the Arp2/3 complex and actin regulation in viral egress based on the identification of a WAVE Regulatory Complex (WRC) Interacting Receptor Sequence (WIRS) in the cytoplasmic tail (CT) of gE. A WIRS-dependent interaction between the gE(CT) and subunits of the WRC was demonstrated by GST-pulldown assay and a role for the Arp2/3 complex in cell-to-cell spread was also observed by plaque assay. Subsequent study of a recombinant HSV-1 gE WIRS-mutant found no significant changes to viral production and release based on growth kinetics studies, or changes to plaque and comet size in various cell types, suggesting no function for the motif in cell-to-cell spread. GFP-Trap pulldown and proximity ligation assays were unable to confirm a WIRS-dependent interaction between gE and the WRC in human cell lines though the WIRS-independent interaction observed *in situ* warrants further study. Confocal microscopy of infected cells of neuronal origin identified no impairment of gE WIRS-mutant HSV-1 anterograde transport along axons. We propose that the identified gE WIRS motif does not function directly in recruitment of the WRC in human cells, in cell-to-cell spread of virus or in anterograde transport along axons. Further studies are needed to understand how HSV-1 manipulates and traverses the actin cytoskeleton and how gE may contribute to these processes in a WIRS-independent manner.

## Introduction

Herpes simplex virus type-1 (HSV-1) lays dormant in approximately 67 % of individuals worldwide [1]. The typical orolabial or genital lesions are of short duration, but infection can cause encephalitis, neonatal herpes and blindness and systemic infections in immunocompromised populations. With no vaccine available, research into new antivirals is critical for managing the increasing incidence of antiviral resistance as well as the impact of virus shedding events. One approach to the discovery of new antivirals is the identification of host-pathogen interfaces necessary for viral replication and spread [2]. Glycoprotein E (gE) is an HSV-1 envelope protein critical for viral egress and cell-to-cell spread. While a large body of research has addressed the roles of gE in transport and cell-to-cell spread, host protein(s) that mediate these functions have yet to be identified [3–9].

To identify host interaction partners involved in gE-regulated egress, we employed a targeted approach aiming to identify and subsequently characterize potential binding motifs present in the cytoplasmic tail (CT) of gE that may play a role in the recruitment of transport pathway regulators. Considering that the actin cytoskeleton forms a structural barrier through which HSV-1 must traffic to escape the cell for exocytosis (reviewed in [10–13]), we hypothesized that egress may function through the regulation of cytoskeletal dynamics.

In 2014, Chen *et al.* identified a motif responsible for recruitment of the WAVE Regulatory Complex (WRC), termed the WRC Interacting Receptor Sequence (WIRS) [14]. The WRC regulates the WAVE-Arp2/3 complex nexus that controls branched F-actin assembly dynamics [15]. The WIRS motif binds a highly conserved surface of the WRC formed by the subunits Sra1 and Abi2 [14]. The WIRS consensus sequence is Φ-x-T/S-F-X-X, where ‘Φ’ prefers bulky hydrophobic residues, ‘x’ represents any residue, and X-X residues represent a pair of amino acids that do not directly bind the WRC but influence binding of the WIRS to the WRC [14]. Mutation studies, whereby the WIRS motif was disrupted by a double alanine substitution of the T/S-F residues of the motif, abolished the interaction between these WIRS-containing ligands and the WRC. Importantly, it was reported that for the multiple WIRS-containing proteins tested, a differential effect on WRC activity towards the Arp2/3 complex after binding was observed, ranging between inhibitory effects, coactivation effects to no effect [14], highlighting the need for functional studies subsequent to motif discovery.

We sought to determine a role for a novel WIRS motif in the gE(CT) in the context of HSV-1 assembly and egress, the first study conducted on a putative WIRS motif in a virus at time of writing. We hypothesized that HSV-1 egress could be regulated by gE(CT) WIRS-dependent recruitment of the WRC to modulate the actin cytoskeleton and mitigate viral egress. We therefore aimed to establish the conservation of the gE WIRS motif among alphaherpesviral gE homologs, identify WIRS-dependent recruitment of the WRC, and characterize the proposed actin regulation pathway hijacked by this virus–host interaction using a combination of phenotypic markers.

Here we demonstrate a WIRS-dependent interaction between gE and members of the WRC by GST-pulldown using rat brain synaptosome lysates as well as a role for the Arp2/3 complex in cell-to-cell spread in HaCaT cells. All subsequent assays in human cells, however, exhibited no gE WIRS-dependent recruitment of the WRC nor any function of the WIRS motif in viral release, cell-to-cell spread or anterograde axonal transport. We therefore propose that the gE(CT) WIRS motif does not function in HSV-1-mediated actin regulation and highlight the need for future studies to understand the biochemical interaction between gE and the WRC, and how the Arp2/3 complex influences viral egress dynamics.

## Methods

Please refer to the Supplementary Methods file (available in the online version of this article) for details on methods used for protein sequence analysis (*in silico*) and recombinant virus generation and characterization.

### Cell lines and growth conditions

HeLa (ATCC CCL-2), HaCaT (provided by Laurence Levy at the INSERM, France), Vero (ATCC CCL-81) and SH-SY5Y (ATCC CRL-2266) cells were regularly maintained and passaged in T75 flasks in DMEM (Lonza) supplemented with 10 % (v/v) foetal bovine serum (FBS), incubated in a humidified 37 °C (5 % CO_2_) atmosphere. Manipulation of HeLa cells was performed with heat-inactivated FBS. SH-SY5Y cells exist as a mixed population of both adherent and suspension cells. Cells in suspension were routinely washed off during regular passaging and not carried through to the next passage. Adherent cell counts were used for seeding in experiments that required SH-SY5Y cells.

#### Differentiation of the SH-SY5Y cell line

Differentiation of SH-SY5Y cells requires a 6 day protocol of retinoic acid treatment preceded by coating of appropriate culture materials with poly-d-lysine (PDL) and laminin to encourage surface attachment. This protocol adapts previously published methods detailed in [16, 17].

Culture surfaces (plastic plates or glass coverslips) were coated with 0.1 mg ml^−1^ poly-d-lysine (PDL, Sigma-Aldrich) in borate buffer (52 mM boric acid, 12.4 mM sodium tetraborate) and incubated for 24 h (37 °C, 5 % CO_2_). PDL was removed and surfaces washed twice with room temperature (RT) sterile water and then coated with 10 µg ml^−1^ laminin (Sigma-Aldrich) for 24 h (37 °C, 5 % CO_2_). Laminin was removed and culture dishes washed twice in RT water. Undifferentiated SH-SY5Y cells were seeded at an appropriate density in an appropriate volume of growth medium and left to settle and attach for 4 h (37 °C, 5 % CO_2_). SH-SY5Y Differentiation Medium (Neurobasal-A Medium (Thermo Fisher Scientific)+100 U ml^−1^ penicillin+100 µg ml^−1^ streptomycin+2 mM l-glutamine+5 ng ml^−1^ brain-derived neurotrophic factor+10 µM all-*trans* retinoic acid) was used to replace seeding medium. Cells were incubated for 6 days (37 °C, 5 % CO_2_) with media changes every 2 days. At day 6, cells were ready for further experimentation. Visual observation of differentiation progression was performed by brightfield microscopy and neuron maturity was confirmed by *in situ* MAP2, synaptophysin and tau expression following fixation and detection for immunofluorescence microscopy. Differentiated cells are referred to herein as dSH-SY5Y cells.

### Phenotypic studies

#### Multi-step growth kinetics

Multi-step growth kinetics were performed in 6-well plates seeded with either 4×10^5^ HeLa cells or 1.2×10^6^ HaCaT cells per well and grown for 16 h (37 °C, 5 % CO_2_). Using a multiplicity of infection (MOI) of 0.001, kinetics assays were performed as described in [18]. Viral titres were determined by plaque assay [19].

In addition, to suppress extracellular virus release and force cell-to-cell spread as the only mechanism of spread possible, another condition included 500 µg ml^−1^ human gamma globulin (HGG) (as a source of human HSV-1 neutralizing antibodies [3]) in DMEM supplemented with 10 % (v/v) FBS and 100 U ml^−1^ penicillin, 100 µg ml^−1^ streptomycin. While difficult to compare specific concentrations of HSV-1 neutralizing antibody to the publication we were attempting to reproduce [3] because of the different sources of HGG and the levels of antibodies present in the population of people whose sera were sampled for product manufacture, 500 µg ml^−1^ was enough to inhibit detectable extracellular virus in HaCaT cells at 24 h after infection at an MOI of 0.001.

#### Infection assays for immunofluorescence microscopy

Synchronized infection was commonly performed for immunofluorescence assays when studying host and/or viral protein distribution at endogenous levels. We used 13 mm glass coverslips in 24-well plates or Lab-Tek II 8-well chamber glass slides (Thermo Fisher Scientific) with 0.7 cm^2^ surface area per well which were seeded with cells to achieve ~60–70 % confluence at the time of infection. Synchronous infection was achieved following a described protocol [20] which lowers the temperature of initial infection to 4 °C to allow for virus/host-cell receptor binding but doesn’t permit viral entry. In this study, HeLa and HaCaT cells were infected at an MOI of 1–3. Because of the fragility of the narrow neurites produced by dSH-SY5Y cells on coated coverslips, synchronous infection of these cells used a modified protocol. Cells were infected at an MOI of 5 made up in SH-SY5Y Differentiation Medium and wash steps were performed with Neurobasal-A medium. Warm infection steps used SH-SY5Y Differentiation Medium again. No virus inactivation step was performed but instead cells were washed twice before fresh warm medium was applied. Following infection, all cells were incubated for 18–24 h at 37 °C (5 % CO_2_) until fixation.

#### Infection assays for GFP-Trap protein studies

HeLa or HaCaT cells were seeded in T75 flasks to reach confluency at 24 h. Cell monolayers were rinsed once with 4 ml DPBS before they were infected at an MOI of 3 in DMEM supplemented with 2 % (v/v) FBS and 100 U ml^−1^ penicillin, 100 µg ml^−1^ streptomycin for 1 h (37 °C, 5 % CO_2_). Viral inoculum was removed, the cells washed once in DPBS, and cells overlaid with 12 ml DMEM supplemented with 10 % (v/v) FBS and left to incubate for a further 23 h. At 24 hpi, cells were lysed and processed for GFP-Trap.

#### Plaque size assay

Plaque size assays were performed as for titration [19] but with confluent monolayers of HaCaT or Vero cells in 6-well plates. Then 5–100 plaque forming units (PFU) of each virus of interest was used to infect cell monolayers and plates were incubated at 37°C for 72 h before overlays were removed and the plaques visualized by crystal violet staining. For Arp2/3 complex inhibitor assays, the overlay was supplemented with cytochalasin D (Sigma-Aldrich), CK-689 (Merck) or CK-666 (Merck) (or vehicle DMSO). Stained wells were imaged with a Canon EOS 5D Mark II camera fitted with a Canon 100 mm USM Macro lens (inhibitor studies) or on a ChemiDoc Touch (Bio-Rad) using acquisition settings for ‘optimal auto-exposure’ Coomassie Protein Stains (recombinant virus studies). Images were black/white inverted and then exported as .tif files for measurement. The horizontal plaque diameter in millimetres was measured using FIJI (version 2.0.0-rc-69/1.52 p [21]) across the widest point of the plaque. Only discrete plaques were measured (overlapping or incomplete plaques at the edge of a well were excluded from analysis). Where different numbers of plaques were measured for each virus across biological replicates, the values were pooled and then randomized based on atmospheric noise calculations [22] and the first 50 values (*n*=50) taken. These values were plotted and statistically analysed.

#### Comet length assay

Comet length assays were performed as per plaque size assays but with a liquid overlay composed of DMEM supplemented with 2 % (v/v) FBS and 100 U ml^−1^ penicillin, 100 µg ml^−1^ streptomycin. Images were captured and data analysed as for plaque assays using *n*=30. Comet lengths (in millimetres) were measured in a straight line from the leading edge of the comet (a completely cleared plaque of lysed cells) to its discernible end (where crystal violet staining returns to background mock-infected levels).

### Protein complex studies

#### GST pulldown

An *Escherichia coli* (* E. coli *) expression system was used to produce large-scale GST-tagged fusion proteins for pulldown assays as previously described [23–25]. The pGEX-5X-1+pU_S_9(CT) has been described previously [24]. pGEX-5X-1+gE(CT) (containing the nucleotide sequence encoding amino acids 447–550 of gE) was generated by PCR amplification of the 17–37 BAC DNA [26] and subsequent ligation into EcoRI/XhoI-linearised pGEX-5X-1 (GE Healthcare Life Sciences). pGEX-5X-1+gE(CT)_AA_ was created by site-directed mutagenesis of pGEX-5X-1+gE(CT) to generate a T530A/F531A mutant protein using primers gE T530A/F531A Forward (5′-CCGCCAGCTCACA**G**CC**GC**TGGATCCGGAAGG-3′) and gE T530A/F531A Reverse (5′-CCTTCCGGATCCA**GC**GG**C**TGTGAGCTGGCGG-3′) and the QuikChange Lightning Site-Directed Mutagenesis Kit (Agilent Technologies). Underlined primer sequence represents the two codons to be mutated, with bold nucleotides representing mutant sequence.

GST-fusion proteins were immobilized on Glutathione Sepharose 4B beads (GE Healthcare Life Sciences) as described previously [24, 25].

Preparation of rat brain synaptosome lysates was performed by Dr Jing Xue at the Children’s Medical Research Institute, Westmead, Sydney, Australia. Rat brain terminal synaptosomes were prepared as described previously [27] and lysed in Synaptosome Lysis Buffer (5 mM Tris (pH 7.5), 100 mM NaCl, 1 % (v/v) Triton X-100, 1 mM EDTA, 1 mM EGTA, 10 µg ml^−1^ leupeptin, 1 mM PMSF, and EDTA-free cOmplete Protease Inhibitor Cocktail).

Binding of lysates was performed as previously described [25] with the following modifications. First, 150 µl of 33 % slurry (=50 µl of beads) was used for each binding experiment. Beads were washed twice in PBS+0.1 % (v/v) Triton-X-100 to remove glycerol buffer (centrifuged at 500 ***g*** for 5 min at 4 °C between washes) before incubation with 1 ml of synaptosome lysate with orbital rotation for 1 h at 4 °C. Beads were centrifuged at 500 ***g*** (4 °C, 5 min) to remove unbound protein-containing supernatant, leaving ~100 µl in which beads were resuspended for transfer to a 0.8 ml Pierce Centrifuge Column (Thermo Fisher Scientific, #89868). Following a triplicate rinse in PBS, protein was eluted by heating beads at 92 °C for 10 min in 100 µl of 2X SDS-PAGE Sample Buffer (Sigma-Aldrich) followed by centrifugation for 30 s at 1000 ***g***. Eluted protein was analysed by SDS-PAGE and immunoblotting.

#### Co-Immunoprecipitation for immunoblot (GFP-Trap)

To perform co-immunoprecipitation of GFP-tagged fusion proteins, the GFP-Trap system from Chromotek was employed. After infection conditions were complete in a T75 flask format, adherent cells were harvested. Cells were washed with ice-cold sterile DPBS prior to lysis. Cells were incubated with 300 µl of GFP-Trap_A Lysis Buffer (10 mM Tris/Cl pH 7.5, 150 mM NaCl, 0.5 mM EDTA, 0.5 % NP-40 (IGEPAL CA-630), 1/100 Mammalian Protease Inhibitor Cocktail (Sigma-Aldrich)) at 4 °C for 30 min with rocking before being scraped into pre-cooled tubes. Cellular debris was pelleted by centrifugation at 20 000 ***g*** (4 °C, 10 min) and the supernatant collected. The rest of the protocol followed manufacturer’s recommendations with the following modifications: sample volumes were adjusted to 500 µl with GFP-Trap_A Dilution Buffer (10 mM Tris/Cl pH 7.5, 150 mM NaCl, 0.5 mM EDTA) and 30 µl of GFP-Trap_A Beads (50 % slurry, Chromotek) was used for each reaction.

### Analysis of protein complexes

#### Sodium dodecyl sulphate-polyacrylamide gel electrophoresis (SDS-PAGE)

Cell lysates and GFP-Trap pulldowns were separated by SDS-PAGE [28] using the Mini PROTEAN Tetra Cell system (Bio-Rad). Gels were run at a constant 200 V in SDS-PAGE Running Buffer (25 mM Tris base, 192 mM glycine, 3.5 mM SDS).

#### Total protein staining

SDS-PAGE gels were stained with SimplyBlue SafeStain (Invitrogen) using manufacturer recommended conditions for 1.5 mm gels. The Odyssey Infrared Imaging System (LI-COR Biosciences; NE, USA) coupled with the ImageStudio acquisition software (version 5.2) was used for detection of total protein using the 700 nm scanning laser.

#### Immunoblot

Subsequent to the one-dimensional separation of protein by SDS-PAGE, gels were equilibrated in SDS-PAGE Transfer Buffer (25 mM Tris base, 192 mM glycine, 20 % (v/v) methanol, pH 8.3) for 5 min at RT. Protein was transferred onto 0.45 µm nitrocellulose membranes (Bio-Rad) using the Mini Trans-Blot Electrophoretic Transfer Cell system (Bio-Rad). Transfer was performed either overnight at a constant 50 mA or for 90 min at a constant 100 V in SDS-PAGE Transfer Buffer.

Indirect protein detection using specific primary antibodies and species-specific secondary antibodies was performed as previously described [29]. As for total protein detection, the Odyssey Infrared Imaging System (LI-COR Biosciences; NE, USA) coupled with the ImageStudio acquisition software (version 5.2) was used for fluorescent secondary antibody detection. Either/both of the 700 nm and 800 nm scanning lasers were used as required for the selected secondary antibody fluorophore conjugate(s) applied to a membrane.

Primary antibodies and working dilutions used for immunoblotting include: anti-CYFIP1 (or anti-Sra1; rabbit polyclonal, 1 : 1100, Novus Biologicals NBP2-16060); anti-NCKAP1 (or anti-Nap1; rabbit polyclonal, 1 : 1000, Novus Biologicals NBP2-19491); anti-Abi2 (goat polyclonal, 1 : 200, Santa Cruz sc-20327); anti-pU_L_19 (mouse monoclonal [DM165], 1 : 5000, from [30]); anti-pU_L_37 (rabbit monospecific, 1 : 5000, kindly provided by Thomas Mettenleiter [31]); anti-pU_L_48 (mouse monoclonal [1-21], 1 : 1000, Santa Cruz sc-7545); anti-pU_S_9 (rabbit polyclonal, 1 : 1000, from [32]); anti-gE (mouse ascites, 1 : 1000, [33]); anti-gD (rabbit polyclonal, 1 : 4000, Abcam ab18610); anti-GFP (FL) (rabbit polyclonal, 1 : 500, Santa Cruz sc-8334); anti-actin (mouse monoclonal [C-2], 1 : 500, Santa Cruz sc-8432).

Secondary antibodies and working dilutions used for immunoblotting include: anti-mouse IgG (H+L) (goat polyclonal, IRDye680RD, 1 : 5000, Odyssey 926–68070); anti-rabbit IgG (H+L) (goat polyclonal, IRDye800CW, 1 : 5000, Odyssey 926–32211); anti-goat (H+L) (donkey polyclonal, IRDye680RD, 1 : 5000, Odyssey 926–68074).

### Immunofluorescence microscopy

#### Antigen detection

Following experimentation, coverslips were washed in DPBS three times. Fixation was performed in 4 % (v/v) paraformaldehyde (in cytoskeletal buffer (10 mM MES, 150 mM NaCl, 5 mM EGTA, 5 mM MgCl2, 5 mM glucose, pH 6.1)) for 15 min at RT before cells were washed in DPBS a further three times. Cells were permeabilized for 5 min at RT in 0.1 % (v/v) Triton X-100 (in cytoskeletal buffer) before another DPBS wash in triplicate.

For all blocking/washing/probing steps, coverslips were incubated with 50 µl droplets of appropriate solution. Cells were blocked with 2 % (v/v) FBS, 1 % (w/v) BSA (in cytoskeletal buffer) for 20 min at RT before cells were probed with primary antibody(ies) diluted in blocking solution for 1 h at RT. Coverslips were washed three times in PBS, and then probed with secondary antibody(ies) (diluted in blocking solution) for 1 h at RT in the dark. Coverslips were washed in triplicate in PBS, rinsed in Milli-Q water and excess liquid removed. Coverslips were mounted on glass slides on a 5 µl droplet of SlowFade Gold Antifade Mountant with DAPI (Invitrogen). Coverslips were cured at RT overnight before they were sealed with clear nail polish and imaged.

For imaging experiments, the following primary antibodies were used: anti-gE (mouse monoclonal [9H3], 1 : 200, Abcam ab6510); anti-pU_L_19 (rabbit polyclonal, 1 : 200, kindly provided by Gary Cohen [34]); anti-alpha1-adaptin (rabbit monoclonal [EPR7572], 1 : 1000, Abcam ab151720); anti-p-ERK (mouse monoclonal [E-4], 1 : 50, Santa Cruz sc-7383); anti-IKAP (rabbit polyclonal, 1 : 50, Santa Cruz sc-8336); anti-CYFIP1 (or anti-Sra1; rabbit polyclonal, 1 : 300, Novus Biologicals NBP2-16060); anti-NCKAP1 (or anti-Nap1; rabbit polyclonal, 1 : 100, Novus Biologicals NBP2-19491); anti-MAP2 (mouse monoclonal, 1 : 400, Boehringer Mannheim 1284959); anti-synaptophysin (mouse ascites, 1 : 500, Sigma Aldrich S5768); anti-tau (mouse monoclonal, 1 : 400, Boehringer Mannheim 1289977). The following secondary antibodies were used: anti-mouse IgG (H+L) (goat polyclonal, Alexa Fluor 488, 1 : 400, Invitrogen A-11001); anti-mouse IgG (H+L) (goat polyclonal, Alexa Fluor 546, 1 : 200, Invitrogen A-11030); anti-mouse IgG (H+L) (goat polyclonal, Alexa Fluor 633, 1 : 500, Invitrogen A-21052); anti-rabbit IgG (H+L) (goat polyclonal, Alexa Fluor 546, 1 : 200, Invitrogen A-11035). Where required, Alexa Fluor 633 Phalloidin (1 : 300, Invitrogen A22284) was included during the secondary antibody step of the method described above.

#### Proximity ligation assay (PLA) detection

To detect interactions by a PLA method, a DuoLink *In Situ* – Fluorescence assay was employed with Duolink probes Anti-Mouse MINUS and Anti-Rabbit PLUS and red-coloured detection reagents [35] (Sigma-Aldrich). Following fixation and permeabilization of cells, primary antibodies of interest were prepared, and the manufacturer’s recommended protocol followed. The ‘Custom’ protocol was used for probing, using the blocking solution and incubation parameters used for primary antibody probing as in ‘Antigen detection’. Slides were stored at −20 °C until imaged.

#### Image acquisition

Cell imaging was performed at the Westmead Scientific Platforms, which are supported by the Westmead Research Hub, the Cancer Institute New South Wales, the National Health and Medical Research Council and the Ian Potter Foundation.

Epifluorescence microscopy was used for imaging PLA assays. Images were captured using an Olympus BX53 Upright Microscope fitted with an Olympus DP80 camera and connected to a computer running cellSens Standard Software (version 1.12, Olympus). Confocal micrographs were routinely acquired on a Leica TCS SP5 II laser scanning confocal microscope (Leica Microsystems, Wetzlar, Germany) with Leica Application Suite Advanced Fluorescence Software (version 2.7.3.9723). Cells were scanned with an HCX PL APO CS 63.0×1.40 OIL objective and z-stack images were collected with an average 0.38–0.8 µm optical depth between steps, with representative z-layers chosen for presentation.

#### Quantitative image analysis

For PLA analysis, PLA signal channels were deconvolved with Huygens Professional version 19.10 (Scientific Volume Imaging, The Netherlands, http://svi.nl), using the CMLE algorithm, with a theoretical PSF, SNR:40 and 50 iterations. Deconvolved images were opened in FIJI (version 2.0.0-rc-69/1.52 p [21]) and individual puncta per cell were counted with the Cell Counter plugin for five fields of view. Where different numbers of cells were measured for each condition, the values were pooled and then randomized based on atmospheric noise calculations [22] and the first 11 values (*n*=11) for HeLa or 32 values (*n*=32) for HaCaT cells plotted (these sample sizes were determined by the condition with the least number of infected cells).

For the analysis of gE distribution in infected dSH-SY5Y cells, a minimum of four fields of view (FOV) from each of three independent replicates were used. Using FIJI, the total number of cells per FOV was manually counted by identifying individual DAPI-stained nuclei. Cells were then categorized as infected based on their pU_L_19 (VP5) staining, but only pU_L_19-positive cells also expressing gE were assessed (see Figs S9 and S10). Of these gE-expressing cells, each was categorized as either: (i) having gE present in both the cell body and along the axon or (ii) having gE present only in the cell body. Data was then represented as the proportion of cells with cell body and axon-localized gE out of all gE-expressing cells. These values were plotted and analysed using an unpaired two-tailed parametric *t*-test. Raw unfiltered images were used for analysis, but the phase images used for representative FOVs in Fig. 9 had their brightness/contrast individually adjusted in FIJI to allow easier visualization of fluorescent signal along the narrow neurites.

### Statistical analysis

**Fig. 1.**
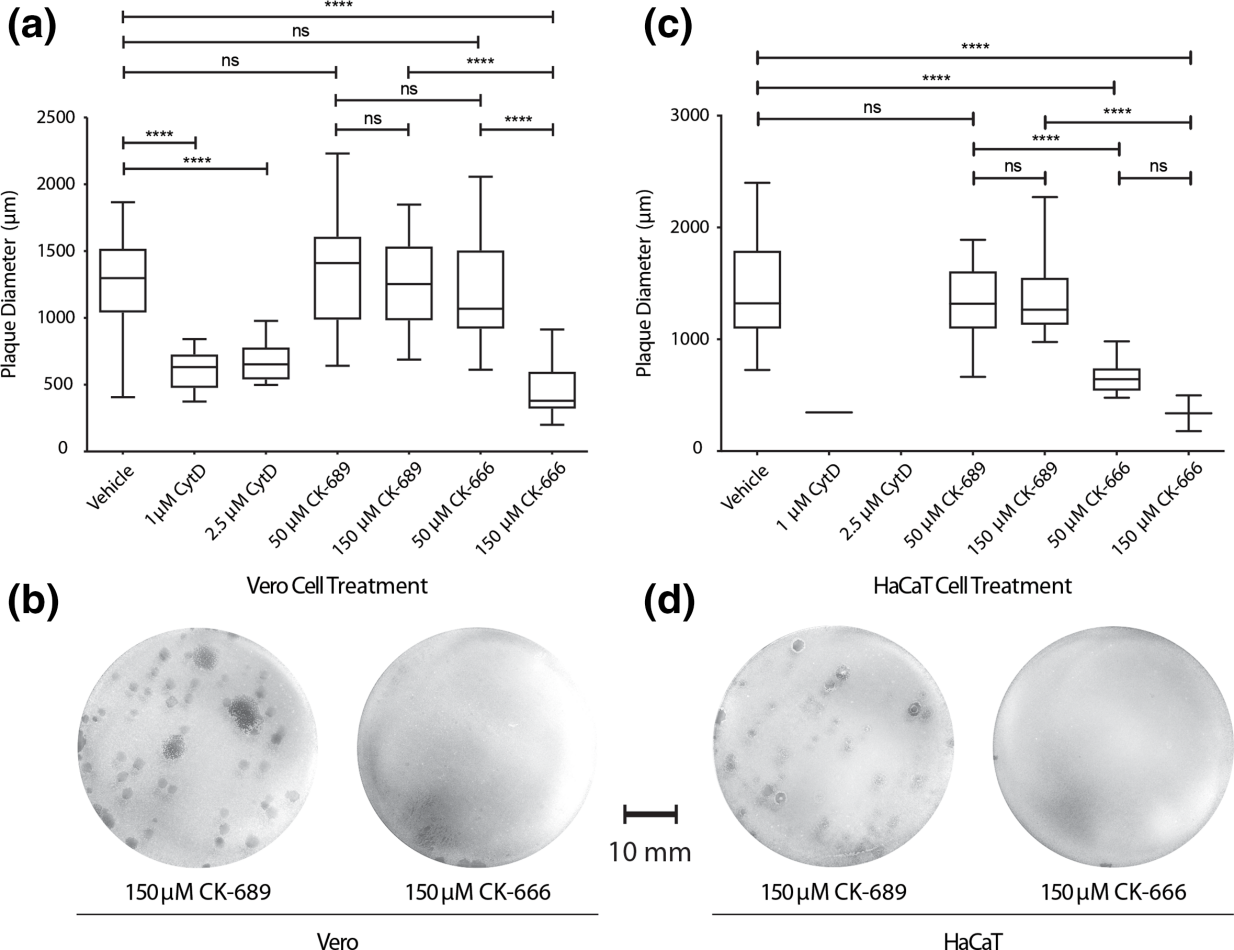
CK-666 reduces HSV-1 plaque size in a dose-dependent manner. Monolayers of Vero (**a**) or HaCaT (**c**) cells were infected with 100 plaque forming units (PFU) of HSV-1 Strain 17 and overlaid with a semi-solid medium under the treatments indicated. After fixation at 72 hpi, plaque diameters were measured along their widest horizontal point. Data from two experimental replicates were pooled (*n*=20 for all except *n*=2 for both 1 µM cytochalasin D (CytD) and 150 µM CK-666 treatments in HaCaT cells). No plaques could be identified in the 2.5 µM CytD treatment of HaCaT cells. Data were analysed using a one-way ANOVA with Tukey’s multiple comparisons test (****P<0.0001; ns, non-significant). (b, d) Representative images of Vero and HaCaT cell monolayers, respectively, treated with the maximum dose of active inhibitor or inactive analogue control.

A one-way analysis of variance (ANOVA) with Tukey’s multiple comparisons test was performed for Fig. 1 in GraphPad Prism 7.0a for Mac, GraphPad Software, San Diego, California USA, www.graphpad.com. Unpaired two-tailed parametric *t*-tests were performed for analysis of Figs 5 and 7–9. Welch’s correction for standard deviation was applied to the *t*-test when column statistics provided within the software identified one or more samples as not passing the Shapiro-Wilk normality test. A *P*<0.05 threshold for statistical significance was set prior to data collection.

## Results

### The Arp2/3 complex functions in HSV-1 cell-to-cell spread

Quantification of viral plaque size is a standard phenotypic assessment of capacity for HSV for cell-to-cell spread. We observed a dose-dependent reduction in viral plaque size in Vero (non-polarized) and HaCaT cells (a polarized epithelial keratinocyte HSV-1 infection model) when infections were performed in the presence of CK-666 (Fig. 1), a specific small molecule inhibitor of Arp2/3 complex-dependent actin filament nucleation [36–38]. CK-689, an inactive CK-666 analogue, had no effect on plaque diameter (Fig. 1). Cytochalasin D (CytD, which non-selectively disrupts actin filaments [39]) had an even more pronounced effect at much lower concentrations, significantly reducing plaque diameter in Vero cells and inhibiting plaque formation in HaCaT cells at 2.5 µM.

### Identification of the gE WIRS motif and conservation within the *Alphaherpesvirinae* subfamily

**Fig. 2.**
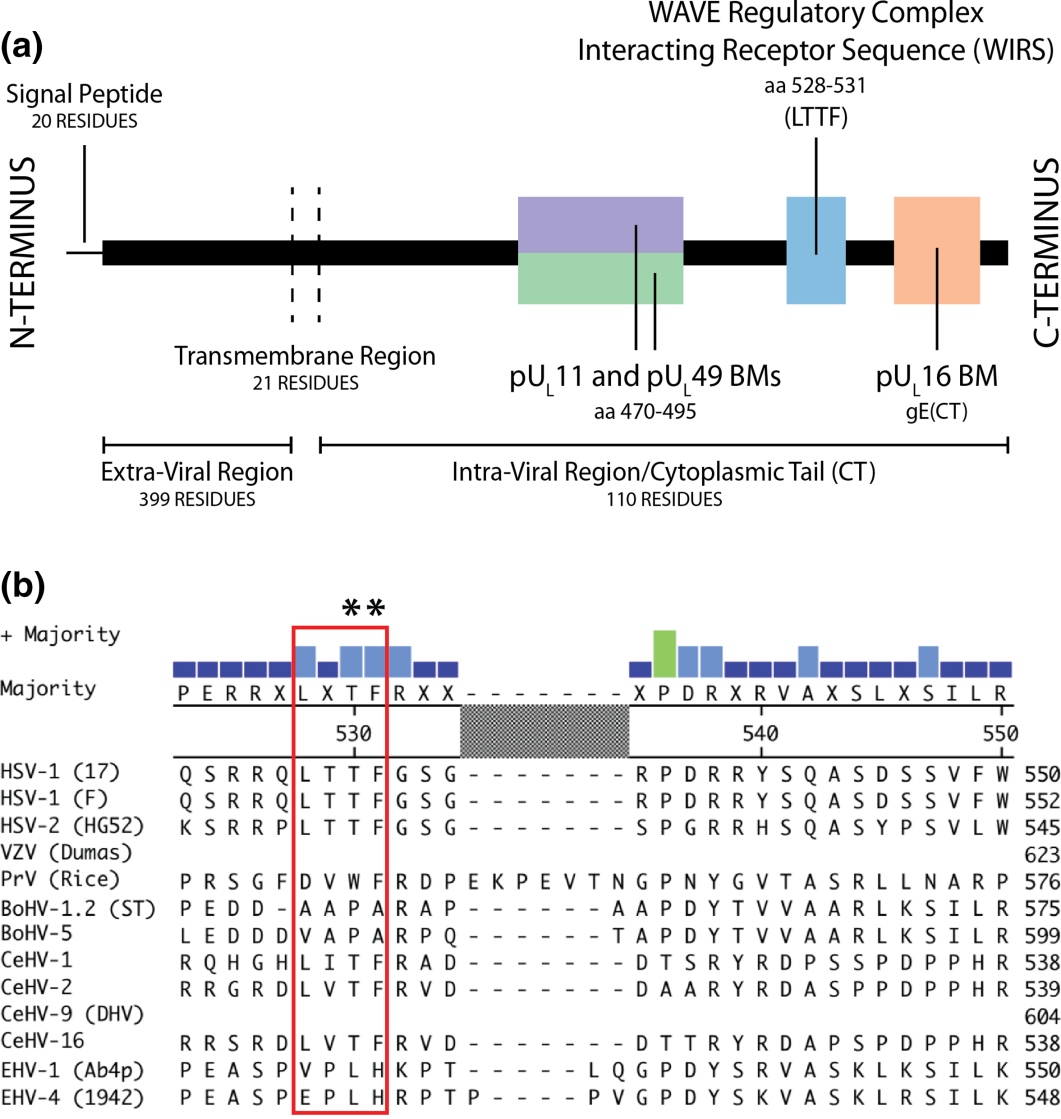
(**a**) A putative WIRS motif is located at the C-terminal end of the cytoplasmic tail of gE. (**b**) The gE WIRS motif is weakly conserved across the *Alphaherpesvirinae* subfamily. ClustalW alignments were generated using Lasergene (version 11.2) MegAlign from DNASTAR. Encoded full-length gE sequences were obtained from the following UniProt Knowledgebase entries (with strains indicated in brackets if available): HSV-1 (17), P04488; HSV-1 (F), Q703F0; HSV-2, P89475; VZV (Dumas), P09259; PrV (pseudorabies virus or suid herpesvirus-1), P08354; BoHV-1 (bovine herpesvirus-1), Q08101; BoHV-5 (bovine herpesvirus-5), Q6X1Z8; CeHV-1 (cercopithecine herpesvirus-1 or simian herpes B virus), P30816; CeHV-2 (cercopithecine herpesvirus-2 or simian agent 8), Q5Y0N5; CeHV-9 (cercopithecine herpesvirus-9), Q04548; CeHV-16 (cercopithecine herpesvirus-16 or herpesvirus papio 2), Q2HWX3; EHV-1 (Ab4p), Q6S6V7; EHV-4 (1942), Q787N9. HSV-1 (17) sequence was used as the amino acid ruler for the consensus sequence, with alignments of all gE homologs displayed between amino acids 523–550 displayed here. VZV (Dumas) and CeHV-9 gE sequences do not align to HSV-1 gE within this region. Consensus residues are shown above alignments and consensus strength displayed as coloured columns (green=8–10 residues match; light blue=6–7 residues match; dark blue=5 or less residues match). The gE WIRS motif is indicated by the red box. Gaps (-) were introduced by the algorithm to maximize alignment. Residues indicated with asterisks represent the amino acids mutated to alanines for mutational studies.

Since only the cytoplasmic regions of viral membrane proteins can interact with host cell binding partners, we interrogated the cytoplasmic sequences of all HSV-1 Strain 17 envelope or envelope-associated proteins for the presence of the WIRS motif consensus sequence Φ-x-T/S-F as described in [14] (using the search string (FMWYIL)X(TS)F) (Table S1). Analysis of the gE protein sequence of HSV-1 Strain 17 identified a potential WIRS motif at amino acids 528–533 (L-T-T-F-G-S; key residues underlined) within the gE(CT) (Fig. 2a). gE was identified as the only HSV-1 membrane protein with a putative WIRS motif present in its cytoplasmic tail surrounded by the required disordered sequence (Fig. S1).

Evolutionary conservation of protein sequence typically indicates an important functional role for key amino acid residues or sequences [40]. We sought to identify how well conserved the gE(CT) WIRS motif is across gE homologs from other mammalian alphaherpesviruses including the human-tropic HSV-2 and VZV and the porcine-tropic pseudorabies virus (PrV) (Fig. 2b). The HSV-1 Strain 17 gE WIRS motif was found not to be globally conserved across the 13 aligned gE homologs but is well conserved between the two human herpes simplex viruses (HSV-1 and HSV-2) and multiple simian viruses (CeHV-1,–2, −16), recalling that the second residue of the consensus motif can handle any substitution [14].

### gE interacts with the WRC in a WIRS-dependent manner by GST-pulldown

**Fig. 3.**
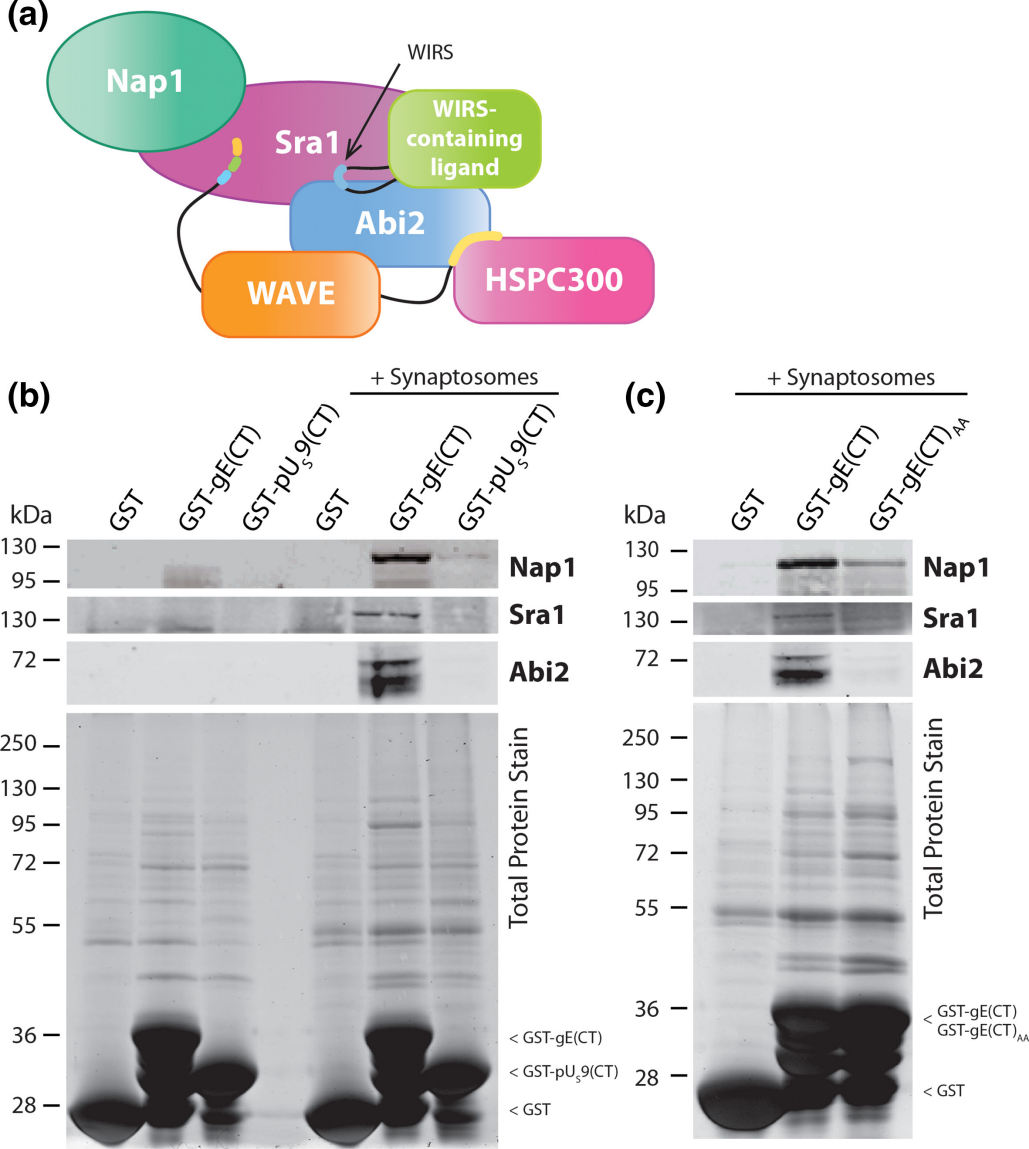
(**a**) The WIRS motif binds a conserved surface formed between WRC subunits Sra1 and Abi2. The WIRS motif of an interacting protein (presented here as a blue segment of a black line representative of a stretch of amino acids within the WIRS-containing ligand) binds to a pocket formed only when the two WRC subcomplexes (the Nap1-Sra1 heterodimer and the WAVE-Abi2-HSPC300 heterotrimer) form the complete heteropentameric complex. HSV-1 gE binds WRC subunits Nap1, Sra1 and Abi2 in a WIRS-dependent manner. (**b, c**) An *in vitro* glutathione *S*-transferase (GST) pulldown was performed using GST-tagged gE(CT) incubated with rat brain synaptosome lysates. GST-gE(CT) pulled down WRC subunits Nap1, Sra1 and Abi2 as confirmed by immunoblot (**b**). T530A/F531A mutation of the WIRS motif (gE(CT)_AA_) demonstrated reduced binding of Nap1, Sra1 and Abi2 (**c**). GST-tagged HSV-1 pU_S_9(CT) was used as a negative control. Note that the total protein stain in **b** includes an empty lane between samples, but the identity of each lane matches the labels aligned above. See supplementary file for original uncropped gel images.

Having identified a putative WIRS in the gE(CT), we observed that gE(CT) was capable of pulling down WRC subunits Nap1, Sra1 and Abi2 (Fig. 3a) by GST pulldown with rat brain synaptosome lysates (Fig. 3b). Mutation of the TF residues to AA (T530A/F531A, recapitulating the WIRS disruption previously published as effective at inhibiting the interaction [14]) was enough to reduce WRC binding (Fig. 3c). The use of rat brain synaptosomes, as an accessible enriched source of the WRC, enabled us to initially identify an interaction between gE and the WRC. Moving forward, we aimed to study this interaction in the context of human cell infection using a whole virus to maintain potential viral co-factors.

### GFP-Trap co-IP of gE-GFP in infected human cell lines

To further study the newly identified gE WIRS motif during human infection, we used a two-stage homologous recombination methodology to introduce genomic alterations into a bacterial artificial chromosome (BAC)-derived HSV-1 Strain 17Syn+parental virus [18, 24, 26, 41]. This technique was used to produce three viruses: a WIRS-mutant (17–37 gE(AA)) as well as C-terminally GFP-tagged versions of both the parental wild-type (17–37 gE-GFP) and the WIRS-mutant (17–37 gE(AA)-GFP) viruses (Fig. S2). Characterization by single-step growth kinetics (Fig. S3) and protein expression assays (Fig. S4) confirmed no biologically significant defects in virus production and release, or changes in representative structural protein expression levels of these recombinant viruses.

**Fig. 4.**
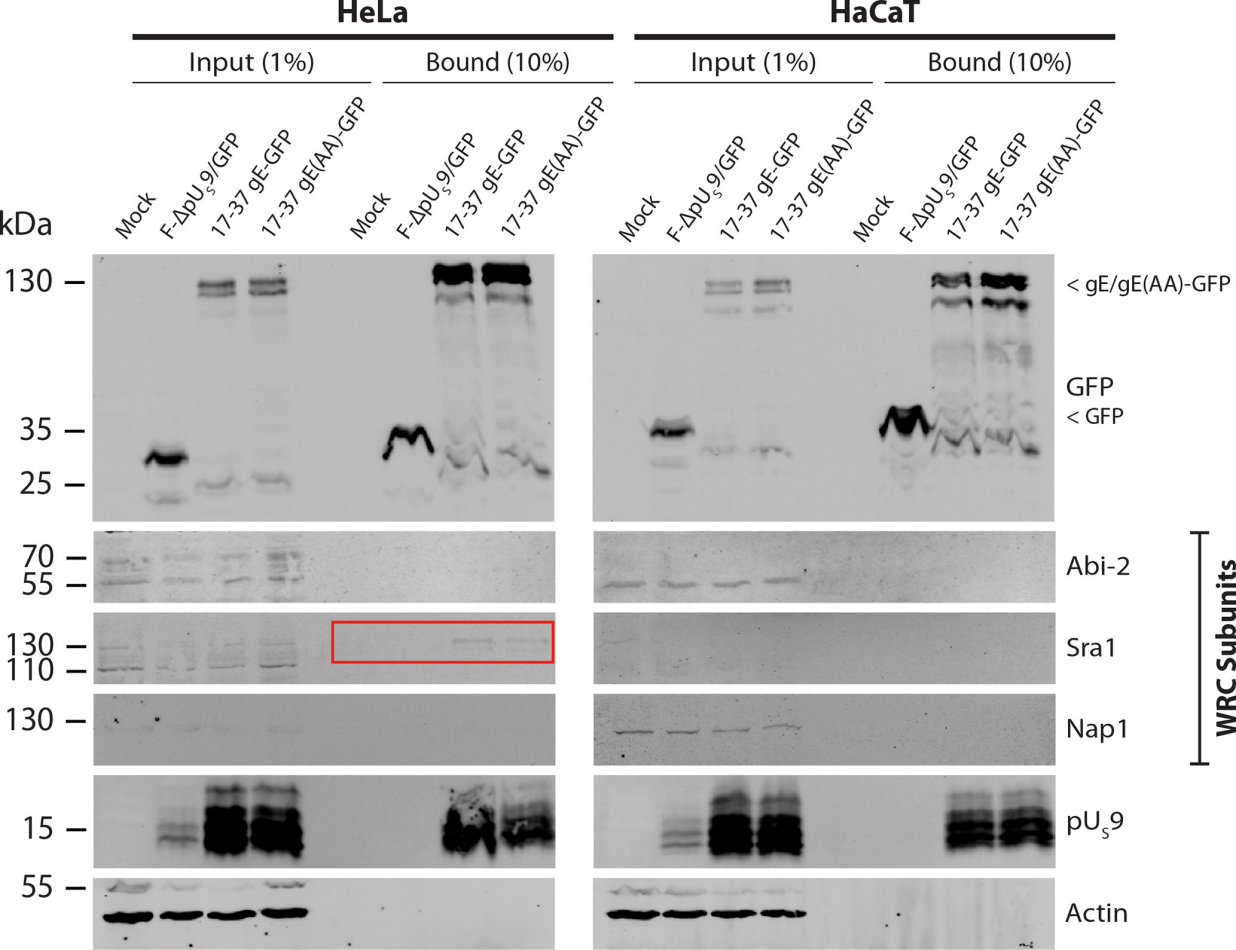
Immunoblot analysis of GFP-Trap co-IP in HeLa and HaCaT cells. Confluent monolayers of HeLa or HaCaT cells in T75 flasks were infected at an MOI of 3 and incubated for 24 h. Cells were lysed and the soluble fraction used as input for GFP-Trap co-immunoprecipitation. The Input and Bound % represent the proportion of the total sample loaded per gel. HSV-1 pU_S_9 was used as a positive control for gE interaction [89]. Highlighted in the red box is the presence of WRC subunit Sra1 (145 kDa) in the bound fraction of both 17–37 gE-GFP and 17–37 gE(AA)-GFP infected lysates. See supplementary file for original uncropped gel images.

The GFP-tagged recombinant viruses (or a control F-ΔpU_S_9/GFP, which replaces the pU_S_9 sequence with GFP to express GFP alone [42]) were used to infect HeLa or HaCaT monolayers (representative of non-polarized epithelial and polarized epithelial keratinocytes, respectively) for 24 h. Infected cell lysates were collected and processed for GFP-Trap co-immunoprecipitation. Comparing the bound fraction to the input fraction in both cell types, we were only able to observe binding of gE to WRC subunit Sra1 in HeLa cells (Fig. 4). The WRC subunits Abi2 and Nap1 were not pulled down by wild-type gE-GFP.

We were unable to observe the same WIRS-dependency observed in the rat brain synaptosome pulldowns in human cells (refer to and compare Figs. 3 and 4). In HeLa cells, gE-GFP and gE(AA)-GFP both pulled-down qualitatively similar amounts of endogenous Sra1. While the Sra1/Abi2 interface in the WRC houses the WIRS motif binding site, we were only able to demonstrate the successful co-IP of Sra1 by gE and not its WRC heterodimer partner Nap1 or its WIRS-coupling partner Abi2.

### Proximity ligation analysis of the gE/WRC interaction *in situ*

As *in vitro* biochemical assays may not always reflect dynamic and transient interactions that take place within a cell, we applied cell biology and *in situ* imaging technologies to study the WIRS motif in the context of the HSV-1 life cycle. Proximity ligation analysis (PLA) coupled with fluorescence microscopy is a technique that is able to assay if proteins of interest are less than 40 nm apart [43]. Here we used PLA technology to examine gE/WRC colocalisation in infected HeLa and HaCaT cell models, comparing 17–37 and 17–37 gE(AA) infections. A series of biological negative and isotype controls were performed to validate the PLA in both uninfected and infected HeLa and HaCaT cells (Fig. S5). A gE/AP-2 colocalisation test was used as a positive control in infected cells and a biological negative control in uninfected cells based on previous findings [44] (Fig. S5).

**Fig. 5.**
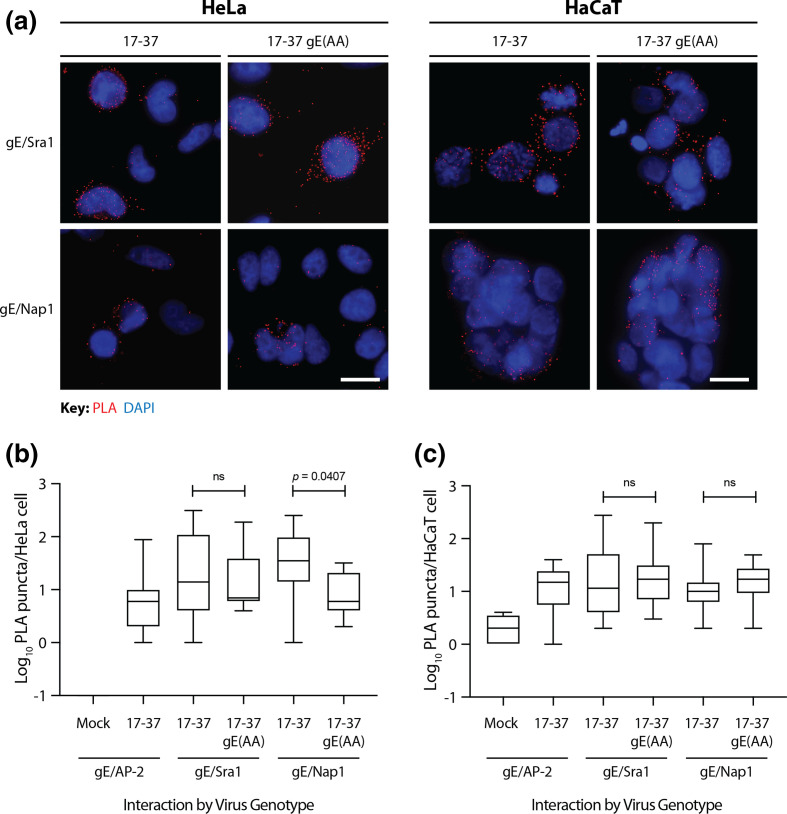
Proximity ligation analysis demonstrates colocalisation of gE with Sra1 and Nap1 is not WIRS-dependent. (**a**) HeLa and HaCaT cells were synchronously infected at an MOI of 2 or 1, respectively, and fixed at 24 h post-infection (hpi). Cells were processed for microscopic analyses using the Duolink *In Situ* – Fluorescence Kit (Red, Mouse and Rabbit). Primary antibodies (rabbit and mouse pairs) were incubated with the cells as indicated before detection was performed using the kit. Images were captured on an Olympus BX53 Upright Microscope fitted with fluorescence filters. Distinct red puncta are representative of colocalisation events. Scale bars represent 20 µm. Images displayed are representative of three independent biological replicates. PLA channel images were deconvolved with Huygens Professional and the number of PLA puncta per infected or mock-infected cell (for the Mock condition) counted for both HeLa (**b**) and HaCaT cells (**c**). For gE/Sra1 and gE/Nap1 infections in HeLa cells, *n*=11; for HaCaT, *n*=32. Two-tailed unpaired parametric *t*-tests with Welch’s correction were performed between the conditions indicated (ns, non-significant).

Compared to controls, an increase in the strength and number of PLA signals (where each red puncta represents an amplified signal from a colocalisation event) was seen for gE/Sra1 and gE/Nap1 in both HeLa and HaCaT cells, providing evidence to support an *in situ* interaction between gE and these WRC subunits (Fig. 5) . Quantitative assessment of PLA signals produced in cells expressing the WIRS-mutant form of the protein, gE(AA), demonstrated no significant change in colocalisation frequencies compared to wild-type in both cell types for WIRS direct interactor Sra1 (Fig. 5b, c), implying that the interaction is not WIRS-dependent. Interestingly, the indirect interactor Nap1 was observed to colocalize with gE(AA) significantly differently to wild-type gE in HeLa cells.

### gE distribution and actin arrangement by confocal microscopy

While we have demonstrated a lack of WIRS-dependence in the putative direct gE/WRC interaction, we sought to observe if gE distribution and/or actin cytoskeleton arrangements were altered in the presence of the WIRS-mutant virus. Analysis of infected HeLa cells at 24 hpi revealed no change in the distribution of gE in the 17–37 gE(AA) virus, providing no evidence for a role of the gE WIRS motif in governing microfilament dynamics (Fig. S6). Infection induced an increase in the number of actin-rich filopodia per cell which was not WIRS-dependent, a phenomenon shown previously to occur as early as 30 min post-infection [45]. Infected cells also maintained the thick stress fibres present in mock-infected cells.

**Fig. 6.**
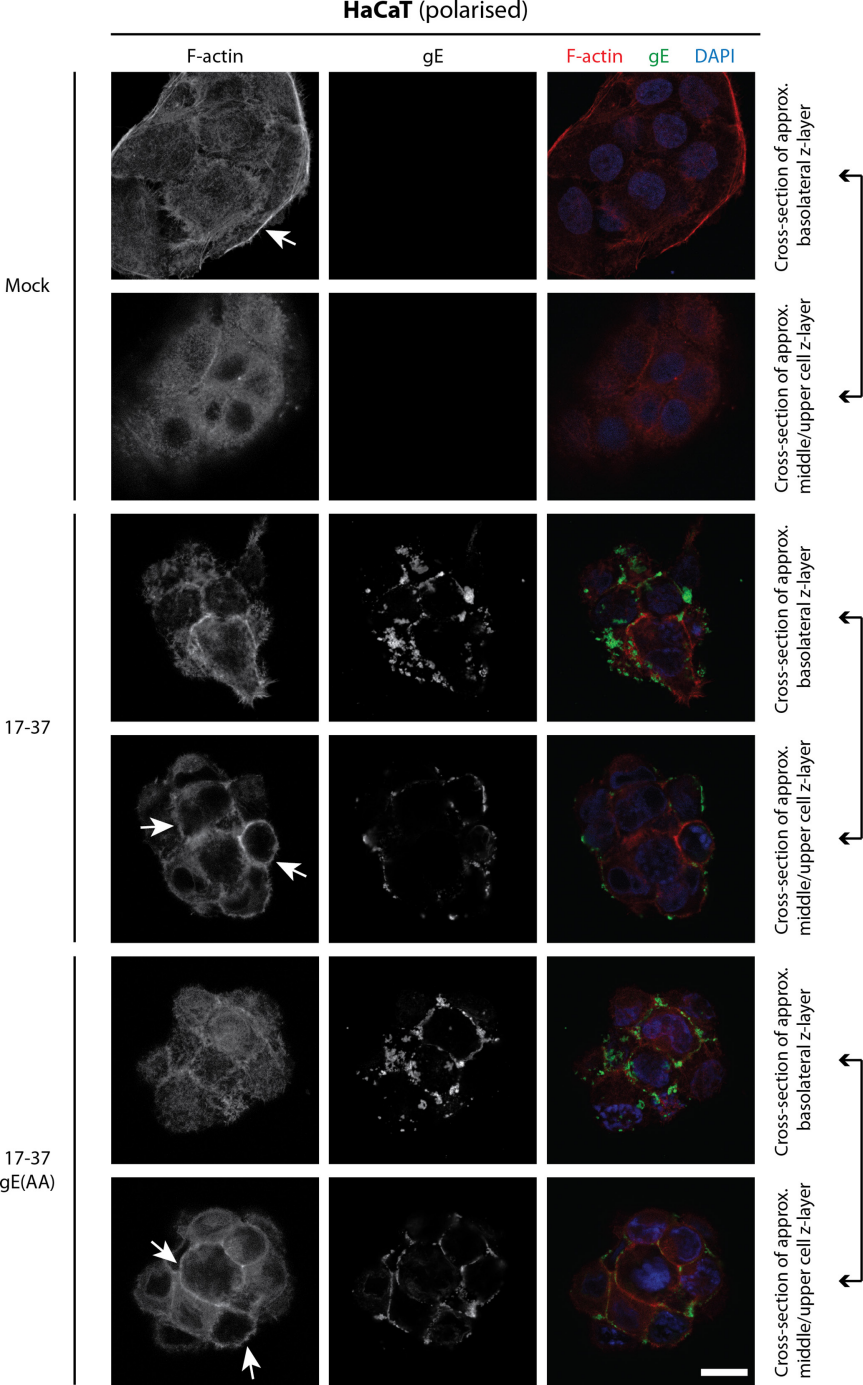
F-actin distribution is unchanged with WIRS-mutant gE. HaCaT cells were synchronously infected with parental 17–37 or recombinant 17–37 gE(AA) viruses at an MOI of 3 for 24 h before fixation. Cells were probed for viral gE and counterstained with phalloidin-AF633 (to stain F-actin; pseudocolored as red) and DAPI. Micrographs were captured on a Leica TCS SP5 II laser scanning confocal microscope. Scale bar represents 20 µm. Images are representative of at least two independent biological replicates. Arrows indicate cortical actin structures.

Actin structures, along with microtubules and intermediate filaments, define cell polarization and are involved in cell spreading and motility via filopodia/lamellipodia formation. We hypothesized that the gE WIRS motif may function as a regulator of actin dynamics in polarized cells and therefore examined gE and F-actin distribution in HaCaT cells (Fig. 6). At 24 hpi with HSV-1, apical actin structures rearrange into cortical actin. However, comparing z-layer images representative of basement membrane and upper cell regions (including part of the apical membrane) in HaCaT cells demonstrated no difference between gE localization nor cortical actin distribution in the presence or absence of an intact WIRS motif. gE was observed at the plasma membrane and cell–cell junctions, independent of the WIRS motif. Moreover, neither gE nor gE(AA) were seen to colocalize with actin structures in either HeLa or HaCaT cells (Fig. 6 and S6).

In polarized HaCaT cells, HSV-1 infection induces robust CPE, with cells clustering more tightly (Fig. 6). Under our assay conditions, infection condensed the thick layer of cortical actin under the plasma membrane, especially towards the apical surface. As for HeLa cells, gE(AA) exhibited no significantly altered distribution compared to wild-type gE. Both gE and gE(AA) were frequently found distributed along cell–cell junctions, suggesting that the WIRS motif plays no role in the gE/gI heterodimer-mediated transfer across cell junctions that has been described previously [3, 46].

### Measurement of viral titres by multi-step growth kinetics

Multi-step growth kinetics assays in HeLa and HaCaT cells were performed to identify any impact of the WIRS mutation on the viral life cycle. Due to the role of gE in egress, we hypothesized that if mutation of the WIRS motif residues limited spread, we would observe a significant decrease in released virus and a concurrent increase in trapped cell-associated virus.

**Fig. 7.**
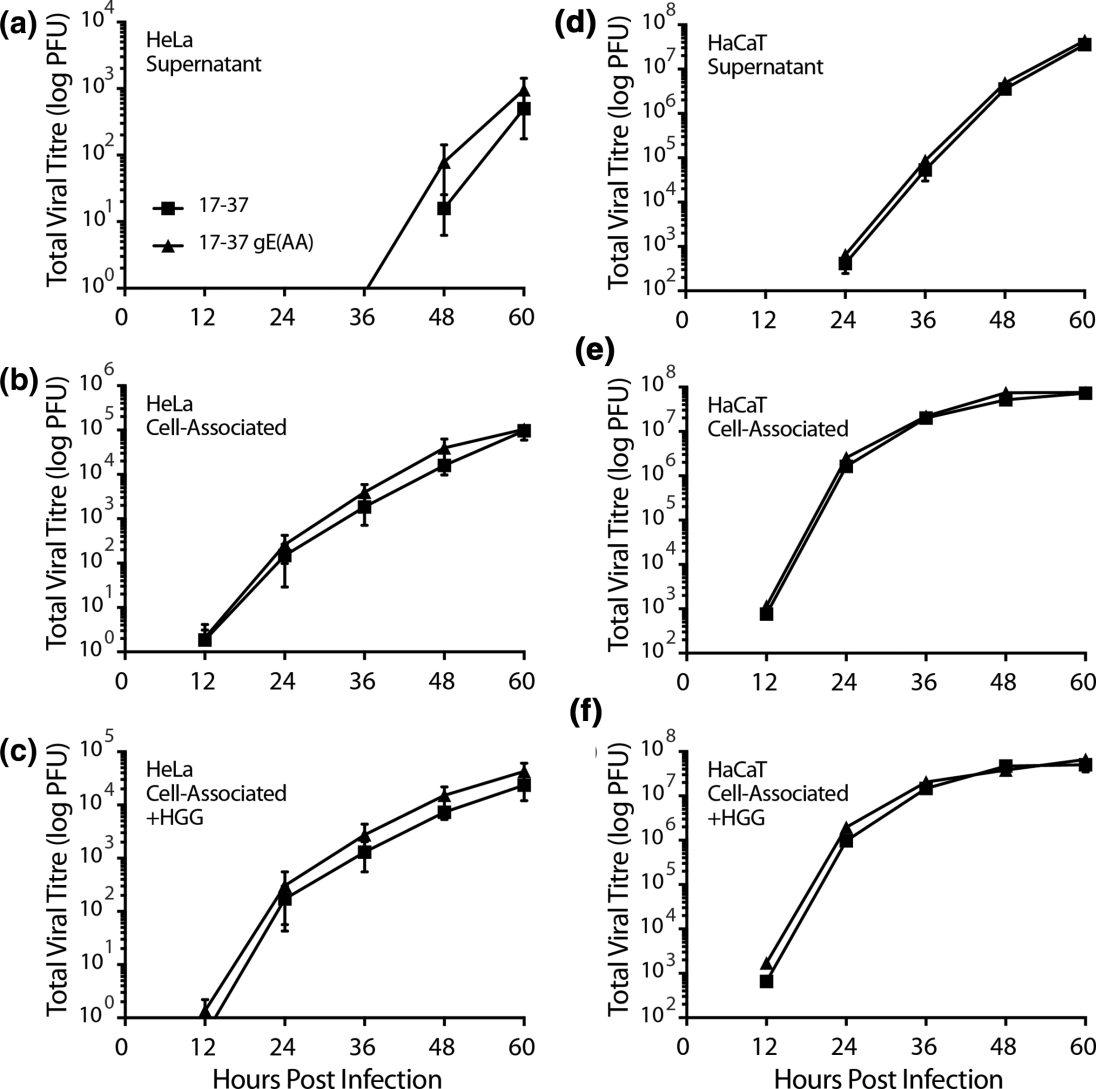
Mutation of the gE(CT) WIRS motif does not affect extracellular virus release or cell-to-cell spread. Confluent monolayers of HeLa or HaCaT cells were infected at an MOI of 0.001 with parental 17–37 (■) or 17–37 gE(AA) (▲) viruses for 1 h. Cells were incubated with Virus Inactivation Buffer for 2 min and wells washed twice with PBS. Cells were overlaid with 2 ml Virus Growth Kinetics Medium (**a, b, d and e**) or 2 ml Virus Growth Kinetics Medium supplemented with 500 µg ml^−1^ HGG (**c, f**) and incubated until harvest. At the times indicated, media was collected and frozen at −80 °C and the cells washed twice with PBS before being scraped into 1 ml fresh medium and frozen at −80 °C. Samples were sonicated and titred by plaque assay on Vero cells. Error bars represent mean±SEM (*n*=3). Two-tailed unpaired parametric *t*-tests with Welch’s correction were performed at each time point.

No significant change in released virus titres was observed in either cell type (Fig. 7a, d). HeLa cells produced approximately 4-log lower extracellular virus titres than HaCaT cells at 60 hpi, with titres below detection limits up to 36 hpi. For both cell types, cell-associated virus titres were similar between 17–37 and 17–37 gE(AA) infections at all time-points (Fig. 7b, e).

Treatment of cells with human gamma globulin (HGG) as a rich source of human anti-herpes neutralizing antibodies was performed in parallel to limit extracellular viral release. Dingwell *et al.* demonstrated a significant effect on cell-associated viral titres for a total gE deletion mutant virus in the presence of HGG, observing up to a 200-fold increase in titres [3]. Therefore, we replicated this approach to test for a phenotype attributable to the more targeted WIRS motif mutation in gE (Fig. 7c, f). At almost all time-points, the untreated infections produced more cell-associated virus than those treated with HGG (ranging up to ~4-fold more for 17–37 and ~2.5-fold more for 17–37 gE(AA) in HeLa cells, and ~1.7-fold more for 17–37 and ~2-fold more for 17–37 gE(AA) in HaCaT cells). No significant effect similar to the 200-fold increase with total gE deletion mutants seen previously [3] was observed for the WIRS-mutant virus.

With 500 µg ml^−1^ HGG in the overlay, no virus was detectable in the supernatant of infected HeLa cells at all time-points but the neutralizing effect of HGG was lost in HaCaT cells by 36 h (Fig. S7). Supernatant virus titres were above the detectable threshold at these timepoints and we conclude that the available neutralizing antibody was saturated by large numbers of virus.

### Observation of cell-to-cell spread and extracellular release

**Fig. 8.**
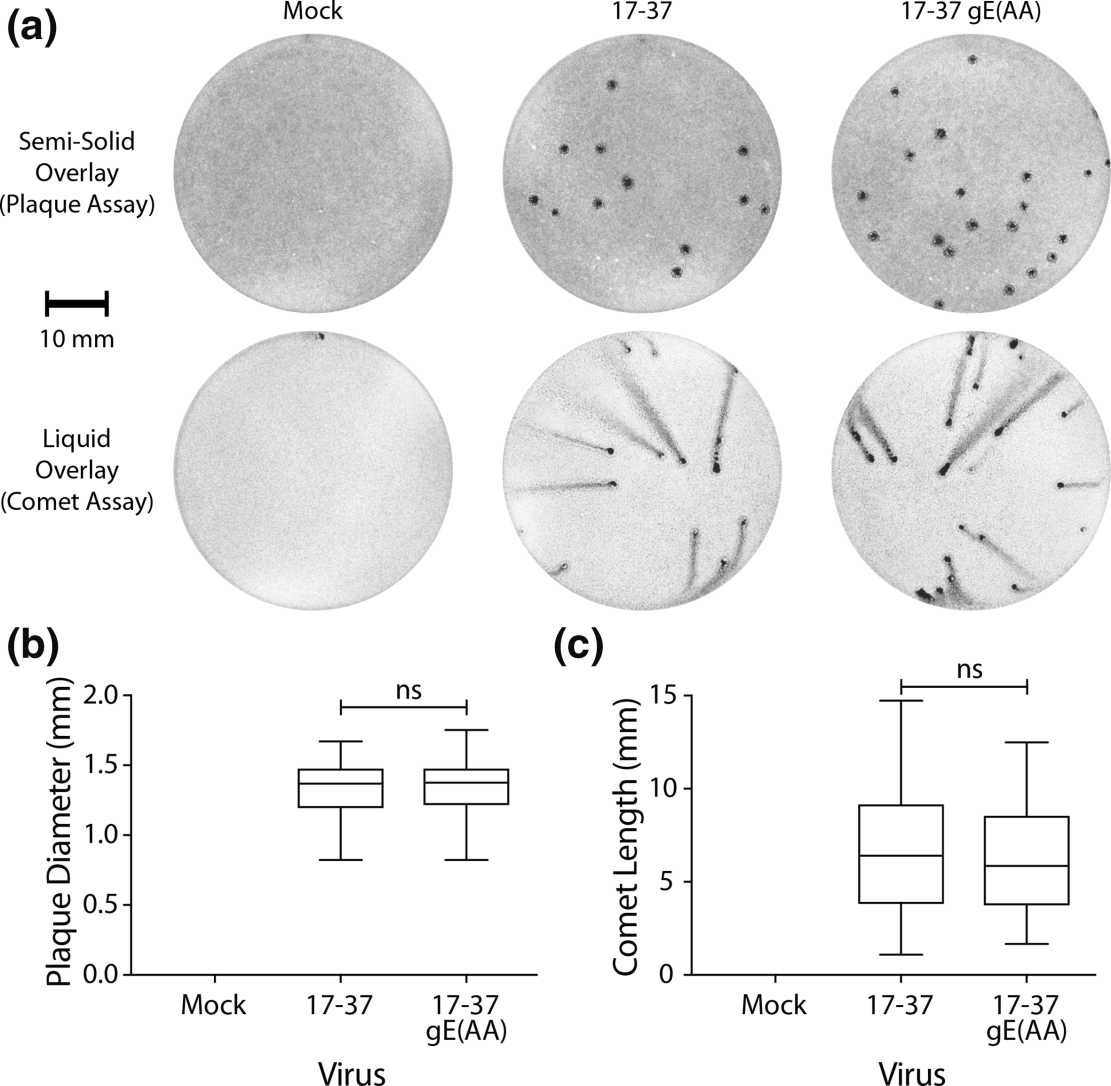
Cell-to-cell spread or extracellular spread of HSV-1 is not WIRS-dependent. (a) In 6-well plates, HaCaT cell monolayers were infected in duplicate with 5–20 PFU of indicated virus for 2 h before cells were overlaid with either a semi-solid carboxymethylcellulose overlay (plaque assay) or a liquid overlay (comet assay). At 72 hpi, overlays were removed, the cells fixed in methanol and plaques/comets visualized by crystal violet staining of the cell monolayer. Images were obtained on a ChemiDoc Touch (Bio-Rad) and plaque diameter (**b**) and comet length (**c**) measured in FIJI. Data for two experimental replicates were pooled (*n*=50 plaques for plaque assay, *n*=30 comets for comet assay) and statistically analysed using a two-tailed unpaired parametric *t*-test with Welch’s correction (ns, non-significant).

Plaque and comet assays were performed to observe spread defects using confluent monolayers of HaCaT cells as HeLa cells do not readily form measurable plaques (Fig. S8a and [47]). Plaque assays are routinely used to determine HSV-1 titres but plaque size measurement (either total plaque area or diameter) can be used as a phenotypic marker of viral spread. In HaCaT cells, 17–37 gE(AA) demonstrated no change in plaque size compared to the wild-type (Fig. 8a, b). The same trend was observed in plaque assays performed in Vero cells (Fig. S8b).

A comet assay, a technique similar in design to a plaque assay but where the semi-solid overlay is replaced with a liquid overlay, was also performed to observe an effect on spread by extracellular mechanisms. These assays are commonly performed in the study of vaccinia virus to study the efficiency of release of extracellular enveloped viruses [48]; the ‘comet’ observed is formed by satellite plaque formation as released progeny virus moves throughout the medium by convection [49]. HaCaT cells produce plaques and comets by 72 hpi under semi-solid and liquid overlays, respectively. Vero cells do not form comets under liquid overlay, instead producing smaller than normal plaques (Fig. S8a). We observed no role of the WIRS motif in extracellular release and spread in HaCaT cells as demonstrated by no statistically significant change in comet length (Fig. 8a, c).

### Assessment of axonal transport in cells of neuronal origin

We then proposed that a requirement for WRC recruitment activity of the putative WIRS motif may be exclusive to cells of neuronal origin, recalling that our initial finding of a WIRS-dependent gE/WRC interaction was seen using lysates of rat brain synaptosomes enriched for a neuronal proteome (Fig. 3). Confocal microscopy was employed to observe transport of virally expressed gE along the axons of human differentiated SH-SY5Y cells (further referred to as dSH-SY5Y cells), a subclone of the SK-N-SH cell line previously used for HSV-1 transport studies [32].

**Fig. 9.**
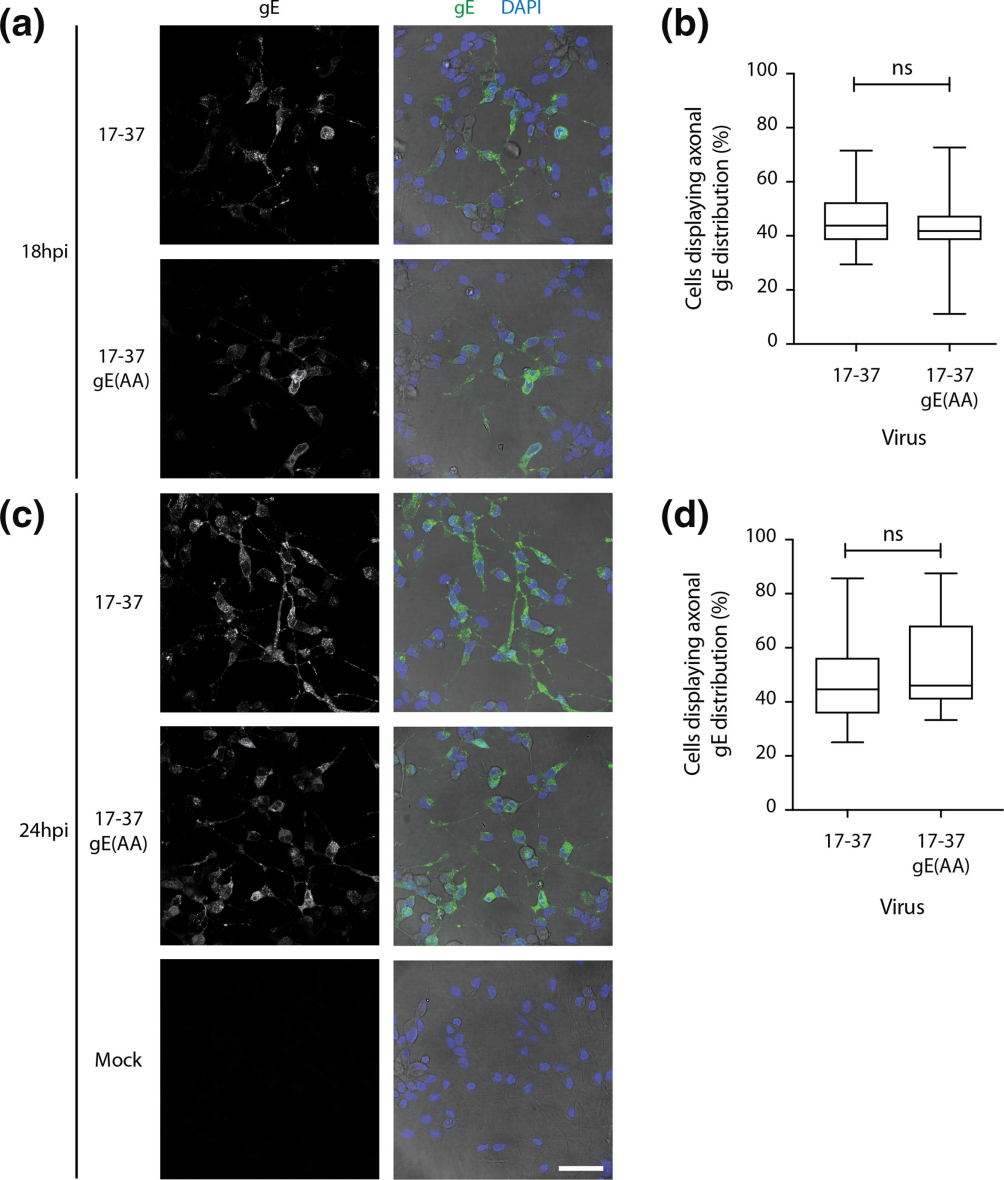
Mutation of the gE WIRS motif does not significantly impact transport along neurites of dSH-SY5Y cells. SH-SY5Y cells were grown on PDL- and laminin-coated coverslips and differentiated over 6 days in the presence of retinoic acid to develop a neuron-like morphology and protein expression pattern. Cells were synchronously infected with parental 17–37 or recombinant 17–37 gE(AA) viruses at an MOI of 5 for 18 (**a**) or 24 h (**c**) before being fixed. Cells were probed for viral gE and counterstained with DAPI to stain nuclei. Micrographs were captured on a Leica TCS SP5 II laser scanning confocal microscope. Scale bar represents 50 µm. A minimum number of four raw and unfiltered fields of view (FOV) for each sample within each of three independent biological replicates were then used to qualify the distribution of gE as cell body only-localized or both cell body and axon-localized, with a percentage calculated and presented for cells expressing axonal gE distribution at each time point (**b, d**). Data was pooled (*n*=14 FOV for 18 hpi, *n*=22 FOV for 17–37 (24 hpi) and *n*=24 FOV for 17–37 gE(AA) (24 hpi)) and was statistically analysed using an unpaired parametric *t*-test with Welch’s correction (ns, non-significant). Error bars represent mean±SEM. For presentation, the phase layers in merged panels were brightness/contrast adjusted in FIJI to present similar colour levels so as not to mask fluorescence signal strength while still portraying axon structures.

SH-SY5Y cells were differentiated until they expressed the markers of neuronal maturation: characterized as redistribution of MAP2 from the cell body to axons; tau expression in axons; and increased synaptophysin expression compared to undifferentiated cells (Fig. S9). Cells were infected with 17–37 or 17–37 gE(AA) for 18 or 24 h (Fig. 9a, c). Infected cells, as defined by co-staining of gE and pU_L_19 (VP5) within the same field of view (accompanying pU_L_19-staining panels are presented in Fig. S10), were compared for their gE distribution: cell body only or both axon and cell body. Cells with both axon- and cell body-localized gE are reported here as a proportion of all gE-expressing cells. At both 18 and 24 hpi, gE(AA) protein was found along a similar proportion of axons as wild-type gE (Fig. 9b, d). No role in transport can be attributed to the gE WIRS motif in dSH-SY5Y cells.

## Discussion

### A role for the Arp2/3 complex in HSV-1 cell-to-cell spread

As HSV-1 must bypass the barrier of the actin cytoskeleton to escape the cell by exocytosis, we were interested in understanding if viral egress functions through dynamic manipulation of cytoskeletal elements. Until now there has been no evidence for Arp2/3 complex-mediated F-actin modulation during HSV-1 infection [50]. Understanding the specific mechanisms and virus–host interactions involved in HSV-1 traversal of the actin cortex is therefore of significant interest.

Using the specific Arp2/3 complex inhibitor CK-666, we found that HSV-1 cell-to-cell spread, a process in which gE plays a critical role, was Arp2/3 complex-dependent (Fig. 1). CK-666 binds to and stabilizes the inactive conformation of the Arp2/3 complex, effectively impeding the movement of key subunits Arp2 and Arp3 into their active positions [36, 51]. Whether redundant pathways are at play because of the ability of CK-666-treated viruses to still spread (albeit in a reduced manner) while CytD (which prevents all actin polymerization) treatment abolished plaque formation is unknown [36–38]. Potentially, since CytD disrupts pre-existing F-actin [39], it can inhibit both actin-based entry and egress of HSV-1, thus blocking the spread of HSV-1 from the initial site of infection. HSV-1 has been shown not to utilize Arp2/3 complex-directed F-actin remodelling during entry by siRNA knockdown studies of Arp2 [50], supporting our evidence that Arp2/3 complex activity is required at later stages in the viral life cycle. Our data implies a crucial function for this complex in cell-to-cell spread, though impacts on earlier stages of the viral life cycle (e.g. gene expression, assembly) cannot be excluded.

### Alphaherpesviral conservation of the gE WIRS motif

In 2014, Chen *et al.* described a protein binding motif termed the WIRS that could recruit and activate the WRC [14]. The multi-subunit nature of the WRC face that the WIRS engages (across both Sra1 and Abi2 subunits of the WRC) indicates specific WIRS-regulation of the complete pentameric WRC only. The authors demonstrated for a variety of WIRS-containing ligands that the motif has variable functionality. It can recruit the WRC to the plasma membrane; it can bind the WRC but do nothing further; or it can bind and flanking sequences trigger inhibition or potentiation of WRC activity. They concluded that WIRS motif binding of the WRC alone is not sufficient for WRC regulation.

The identified WIRS motif in HSV-1 gE was weakly conserved across mammalian alphaherpesviruses (Fig. 2). Complete conservation of the key WIRS motif residues (L-T-T-F-G-S, key residues underlined) was identified in the closely related primate-tropic simplexviruses: HSV-1, HSV-2, CeHV-1, CeHV-2 and CeHV-16 [52]. Non-human primate herpesviruses are much more genetically similar to human herpesviruses than other mammalian herpesviruses [53, 54] and while gE homologs across the viruses compared here all function in cell-to-cell spread [55], these results suggest a uniquely evolved putative binding domain. As gE is predominantly found bound to its dimeric partner gI and performs many of its functions as part of this dimer [3, 56], the lack of a WIRS motif in gI indicates that this putative function could only be mediated by gE.

Interestingly, as VZV gE lacks a WIRS motif in its small cytoplasmic tail (64 aa of a total 623 aa protein, compared to a 110 residue CT for HSV-1 gE), it likely functions differently to HSV-1 gE in cell-to-cell spread. Evidence suggests that VZV gE enhances the formation of intercellular junctions between polarized epithelial cells with recent studies showing this is dependent on its extra-viral insulin degrading enzyme binding domain [57–59].

With gE functioning in HSV-1 egress and cell-to-cell spread [3–5, 7, 8, 46, 60] and initial evidence from GST pulldowns that gE binds WRC subunits in a WIRS-dependent manner (Fig. 3), we hypothesized that gE could be hijacking the Arp2/3 complex via the WRC to remodel the actin cytoskeleton to facilitate egress.

### gE does not interact with the WRC in a WIRS-dependent manner in human cells

A domain located within residues 235–264 of the extra-viral domain of gE is required for the gE/gI complex to form [61], and thus manipulation of the WIRS motif in this study should have no impact on gE/gI complex functions, though gI was not examined in this study. Similarly, manipulation of the WIRS motif should not impair binding of tegument proteins pU_L_11, pU_L_49 or pU_L_16 (which thus far have only been mapped to the gE(CT) [62, 63]), though further study is needed to confirm this. This is crucial because the native function of gE requires the assembly of direct interactors pU_L_11 and pU_L_16 and indirect partner pU_L_21 to its CT [64]. The binding domain on gE required for its interaction with pU_L_51 is yet to be confirmed [65, 66], so the impact of gE WIRS mutation on the gE/pU_L_51/pU_L_7 complex is currently unclear.

The generation of recombinant WIRS-mutant and GFP-tagged gE viruses enabled us to investigate a role for the WRC in human cells of biological relevance to HSV-1 infection. Of the three WRC subunits assayed, including direct interactors of the WIRS Sra1 and Abi2 and indirect interactor Nap1, only Sra1 was seen to be co-immunoprecipitated by gE-GFP (Fig. 4). This occurred only in HeLa cells and in a WIRS-independent manner, which contrasted with our GST pulldown with synaptosome lysate findings which used only gE(CT) in the absence of other viral proteins. The extensive interactions formed between dimer partners Sra1 and Nap1 may have been lost under the stringency of these wash conditions [67], but it is more likely a sensitivity issue: HeLa and HaCaT cells may express lower levels of WRC proteins than the synaptosomes and/or the synaptosome lysates were more concentrated. The fact that only the cytoplasmic tail of gE, in the absence of other viral proteins, was used for the GST-pulldowns could have been a contributing factor since this did not provide the most biologically relevant conditions.

By PLA, we clearly demonstrated that, *in situ*, gE colocalizes closely with WRC subunits Sra1 and Nap1 but the interaction is not WIRS-dependent, consistent with the GFP-Trap results (Fig. 5). Since the canonical WIRS binds to the conserved WRC face formed between Sra1 and Abi2, the PLA was designed to assess loss of a direct interactor (Sra1) as well as the indirect interactor (Nap1) which only come together in the active WRC [67]. Though the preliminary GST pulldown and PLA data agree on a wild-type gE/WRC interaction, our data demonstrate that gE binds to the WRC in a WIRS-independent manner *in situ* in HeLa and HaCaT cells and that the assay conditions used for GFP-Trap were not sensitive enough to detect the indirect gE/Nap1 interaction. Determining how gE interacts with the WRC if not via the WIRS motif is worth further investigation, especially if linked to a role for the Arp2/3 complex. The contribution of other viral protein partners may be necessary, though none of the HSV-1 membrane proteins were found to possess WIRS motifs (Table S1), an observation which requires further study.

We further confirmed by confocal microscopy that the WIRS motif has no impact on actin regulation in HeLa or HaCaT cells (Fig. 6 and S6). Exposure to HSV-1 induced filopodia formation in non-polarized HeLa cells, a phenomenon that has been demonstrated to support entry of incoming virions [68, 69]. The actin stress fibres present in mock-infected cells were maintained in infected cells, and the pattern of actin distribution was no different when the gE(CT) WIRS motif is mutated. gE localization, too, was unchanged, existing in a punctate cytoplasmic distribution.

Additionally, there was no evidence of colocalisation between gE and F-actin in both cell types, providing no evidence for an association of gE with F-actin. In studies of the betaherpesvirus human cytomegalovirus (HCMV), the association of myosin Va, the HCMV major capsid protein and F-actin within the nucleus suggests that actin motors are used for intranuclear HCMV capsid motility [70]. While nuclear actin filaments are also induced during PrV and HSV-1 infection [71] and actin is incorporated into mature HSV-1 virions [72] (suggesting a role in viral assembly), subsequent work by the same group demonstrated that siRNA-mediated knockdown of endogenous actin did not significantly impact HSV-1 virion production [73]. Further work is needed to unravel the contribution of F-actin within the nucleus to the HSV-1 life cycle.

While it is understood that gE functions in cell-to-cell spread, we have provided evidence that the gE(CT) WIRS motif alone is not responsible for proposed WRC recruitment in HeLa or HaCaT cells and plays no function in actin modulation. HCMV pU_L_135 was recently shown to remodel the actin cytoskeleton via the WRC [74], but our analysis of the protein sequence did not identify a putative WIRS motif (data not shown). Stanton *et al.* identified a sequence of residues (amino acids (aa) 169–206) that was required for binding directly to Abi1/Abi2 which enabled recruitment of the WRC for disassembly of the actin framework, facilitating restructuring of the cytoskeleton to evade natural killer cell recognition [74]. This betaherpesviral protein binds and activates the WRC in a WIRS-independent manner; we speculate that the recruitment of the WRC by gE observed in our study may require one or more bridging partners.

As the gE(CT) is only 110 amino acids long, contains multiple binding domains required for interactions with other viral proteins, and is the only domain of the protein accessible to host cell cytoplasmic protein partners, it is possible that the residues thus far described as a putative WIRS motif form part of a larger as-yet-undefined binding domain for regulators of intracellular transport. For example, threonine residues within identified WIRS motifs can undergo phosphorylation (a common activation marker) [14] and there is evidence that the gE(CT) is highly phosphorylated during *in vitro* infection studies both in the presence and absence of viral mediators (e.g. virally-encoded kinases like pU_L_13) [75, 76].

### Non-actin-based studies for viral release and cell-to-cell spread

Quantification of plaque size and multi-step viral growth kinetics are commonly used to evaluate spread phenotypes of recombinant HSV-1. Whole protein deletion mutant viruses for either gE (gE^-^) or gI (gI^-^) release 10–20-fold less virus than parental into the supernatant, and produce 5- and 10-fold less cell-associated virus, respectively [3]. Forcing only cell-to-cell lateral spread by the addition of a source of human anti-herpes neutralizing antibodies in the form of human gamma globulin, Dingwell *et al.* demonstrated a much greater difference (up to 100–200-fold less) for these deletion viruses compared to parental [3]. Conclusively, each of these proteins contributes to cell-to-cell spread. Our investigations applied the same approach for the 17–37 gE(AA) mutant virus in HeLa and HaCaT cells, identifying no biologically significant change in viral titres, both extracellular and cell-associated (Fig. 7). In the presence of HGG, the 17–37 gE(AA) virus was no less successful at producing cell-associated virus than the parental, indicating there was no deficiency in lateral viral spread that could be attributed to the two residues mutated in the WIRS motif.

Further work with gE- and gI- viruses demonstrated reduced plaque sizes in human fibroblasts [3] and in HaCaT cells tested with the gE- virus [6]. In HaCaT cells, a CT deletion mutant (gEΔCT) produces small plaques resembling gE- plaques, suggesting the CT is responsible for cell-to-cell spread [6], a mechanism which appears to compete with apical release of progeny virus from Vero cells [66]. The extra-viral domain of gE alone is sufficient for accumulation at the cell–cell junctions but not for cell-to-cell spread, further confirming the requirement of the gE(CT). Our test of the WIRS-mutant virus failed to demonstrate a function of the motif in cell-to-cell spread in HaCaT cells, with no significant change in plaque size (Fig. 8).

Even using quantitative liquid overlay comet length assays [77], no change in extracellular viral spread was observed (Fig. 8). Comet assays are not typically used in extracellular release studies of HSV-1 but plaque assays performed with liquid overlays containing neutralizing antibodies have been described [78]. Our work presents the first comet assay study of HSV-1 in HaCaT cells though similar liquid-overlay methods have recently been described using BSC-1, SKOV3 and HCT116 cells to observe HSV-1 extracellular spread [79]. Interestingly, different strains have different spread phenotypes: in our study, we found that BAC-derived Strain 17 virus cannot form comets in Vero cells but Jones *et al.* demonstrate large comets for strain SC16 and small (but observable) comets for non-BAC-derived Strain 17 viruses in Vero cells [79].

To this point, all studies on the gE(CT) WIRS used epithelial cells, but the initial interaction was determined using rat brain synaptosome lysates, derived from neurons that express WRC components (and other upstream/downstream effectors) in a different pattern than HeLa and HaCaT [80]. The gE(CT) functions in axonal transport of HSV-1 in neuronal cells [32, 81] where different HSV-1 effectors are at play [11, 12]. Therefore, we analysed gE transport in dSH-SY5Y cells to determine if there was a neuron-specific transport defect for the WIRS-mutant virus. Using an approach similar to that described for differentiated SK-N-SH neurons (from which SH-SY5Y cells were subcloned) [32], we observed that at both 18 and 24 hpi there was no significant change in axonal transport for the 17–37 gE(AA) virus and thus concluded that the WIRS motif has no function in anterograde axonal transport, either (Fig. 9).

### Future directions

Given that HSV-1 appears to require the Arp2/3 complex for cell-to-cell spread, but that no viral membrane protein other than gE possesses a putative WIRS motif, a series of truncations of the gE(CT) could be used to determine which residues within the CT contribute to the WIRS-independent gE/WRC interaction observed, by both PLA and pulldown. Future studies could also investigate the tegument proteins that surround a nascent virion between the TGN and the plasma membrane for the ability to regulate the WRC or directly engage the Arp2/3 complex. Tegument proteins anchor the tegument layer to the cytoplasmic tails of envelope proteins, including those that bind gE (pU_L_11, pU_L_16 and pU_L_49). It is feasible that these proteins bridge a gE/WRC interaction to regulate the final trafficking of virus to the plasma membrane where gE can then function in cell-to-cell spread, which could explain why gE and the WRC were found to colocalize in a WIRS-independent manner by PLA.

Furthermore, the molecular motors myosin IIA and IIB have been implicated in HSV-1 entry and recruitment of myosin Va is responsible for HSV-1 secretion into the supernatant and for adequate surface expression of gM [82–84], though myosins are not involved in nuclear capsid motility [85]; perhaps gE engages myosins to facilitate egress, a hypothesis that would require further study.

With advances in technologies, however, affinity purification coupled with mass spectrometry is a relatively simple and efficient way to identify the interactome of target proteins (i.e. all proteins that interact with the target) [86]. This approach has been used in the alphaherpesvirus field to identify the interactomes of PrV pU_S_9 and gE [87, 88] but this has yet to be extended to HSV-1 gE. The next step in determining the host partners of HSV-1 gE as it functions in egress and cell-to-cell spread will be to identify its interactome in cell types of biological relevance and subsequently interrogate the interactome for proteins involved in transport pathways and validate them further by mutational studies. Such studies are underway in our laboratory and may lead to the identification of critical virus-host interactions (novel antiviral targets) for future therapy development.

### Conclusions

In this study, we aimed to elucidate the role of a putative WIRS motif in the cytoplasmic tail of HSV-1 gE. Having demonstrated a role for Arp2/3 complex-dependent actin dynamics in cell-to-cell spread, we sought to better understand the virus-host interaction partners involved. We conclude that residues 530–531 of the gE(CT) (forming part of the aa528-533 ‘L-T-T-F-G-S’ WIRS motif) are not required for gE binding of the WRC in human epithelial cells and do not contribute to actin modulation. We also found no impact of these residues on viral production, release or cell-to-cell spread, nor anterograde axonal transport.

## Supplementary Data

Supplementary material 1
