# Supplementary material for: A putative WAVE regulatory complex (WRC) interacting receptor sequence (WIRS) in the cytoplasmic tail of HSV-1 gE does not function in WRC recruitment or neuronal transport
Source: Access Microbiol. 2021 Mar 4;3(3):000206. doi: 10.1099/acmi.0.000206 (PMC8209697; doi:10.1099/acmi.0.000206)

A putative WAVE regulatory complex (WRC) interacting receptor sequence (WIRS) in the cytoplasmic tail of HSV-1 gE does not function in WRC recruitment or neuronal transport

Christopher E. Denes, Timothy P. Newsome, Monica Miranda-Saksena, Anthony L. Cunningham, and Russell J. Diefenbach

Supplementary File Table of Contents

Supplementary Methods 2

1. Protein Sequence Analysis (in silico) 2
1.1 Protein Motif Search 2
1.2 Protein Secondary Structure Prediction 2
1.3 Multiple Sequence Alignment 2
2. Recombinant Virus Generation 2
3. Recombinant Virus Characterization 3
3.1 Isolation and Screening of Viral DNA 3
3.2 Single-Step Growth Kinetics 4
3.3 Viral Protein Expression 4
Supplementary Methods References 5

Supplementary Data 6

Table S1. HSV-1 membrane protein WIRS motif consensus analysis. 6
Figure S1. Secondary structure and disordered region prediction of the HSV-1 gE protein sequence. 7
Figure S2. Schematic representation of recombinant BAC-derived HSV-1. 8
Figure S3. Recombinant viruses demonstrate wild-type growth kinetics. 9
Figure S4. Immunoblot characterization of recombinant 17-37 HSV-1 protein production. 10
Figure S5. Proximity ligation analysis controls demonstrate low-level background of colocalizing signals in HeLa and HaCaT cells. 11
Figure S6. F-actin distribution is unchanged with WIRS-mutant gE. 12
Figure S7. HGG neutralization of released virus in HeLa and HaCaT cells at 500 μg/mL. 13
Figure S8. HSV-1 plaque and comet phenotypes in various cell types. 14
Figure S9. Morphological appearance of differentiated SH-SY5Y cells and expression of markers of cell maturity. 15
Figure S10. Supplementary panels for Figure 9 regarding gE/gE(AA) transport along neurites of dSH-SY5Y cells. 16

Original Uncropped Gel Images 17

For Figure 3 17
For Figure 4 18
For Figure S4 20

Supplementary Methods

1. Protein Sequence Analysis (in silico)

1.1 Protein Motif Search

Protein sequences for each of the membrane proteins encoded by the HSV-1 Strain 17 genome were downloaded from UniProt (https://www.uniprot.org) (1). SnapGene software (Version 3.0.3, GSL Biotech LLC) was used to search for consensus motifs. The WIRS consensus sequence ΦX(TS)F was searched for using a search string of (FMWYIL)X(TS)F (2).

1.2 Protein Secondary Structure Prediction

To further confirm the sequence conformed to the prescribed consensus motif published by Chen et al. (2), the 550 amino acid protein sequence was subjected to secondary structure and disordered region prediction using the PSIPRED Workbench server at http://bioinf.cs.ucl.ac.uk/psipred/ (University College London Department of Computer Science: Bioinformatics Group) (3) with simultaneous application of PSIPRED 4.0 and DISOPRED3 analyses using default parameters (4, 5). The Φ-x-T/S-F-X-X consensus is required to be surrounded by disordered protein sequence (2).

1.3 Multiple Sequence Alignment

To align the protein sequences of gE homologs from multiple mammalian-tropic alphaherpesviruses, sequence files were downloaded from the UniProt Knowledgebase as .pro files and aligned in Lasergene MegAlign (Version 11.2) from DNASTAR using the ClustalW algorithm. Simultaneous production of a phylogenetic tree was performed within the MegAlign software.

2. Recombinant Virus Generation

The recombinant viruses HSV-1(17-37)-$gE_{TF/AA}$, HSV-1(17-37)-gE-GFP and HSV-1(17-37)-$gE_{TF/AA}$-GFP (Table S2) were generated using homologous recombination and *galK* selection/counterselection utilizing a bacterial artificial chromosome (BAC)-cloned HSV-1 Strain 17 genome (17-37, kindly provided by David Leib (6)) maintained in *E. coli* SW102 cells as described previously (7-10). Simplified names (Table S2) are used throughout this study.

Table S2. List of BAC-derived viruses generated in this study.

Formal Name	Description	Simplified Name*
HSV-1(17-37)-$gE_{TF/AA}$	HSV-1(17-37) with a mutant WIRS motif sequence in gE (6 base-pair mutation of ACCTTT → GCCGCT in the HSV-1 Strain 17 US8 sequence encodes a T530A/F531A mutation at the protein level).	17-37 gE(AA)
HSV-1(17-37)-gE-GFP	HSV-1(17-37) with a C-terminal EGFP tag on gE separated by a linker.	17-37 gE-GFP
HSV-1(17-37)-$gE_{TF/AA}$-GFP	HSV-1(17-37)-gE-GFP with a mutant WIRS motif sequence in gE (6 base-pair mutation of ACCTTT → GCCGCT in the HSV-1 Strain 17 US8 sequence as above).	17-37 gE(AA)-GFP

* Hyphens have been used to indicate a C-terminal GFP-fusion tag where described.

First stage recombination cassettes were generated using primers gEF-galK/gER-galK for mutation of the gE WIRS motif (T530A/F531A) or GFP-gE(galK)F/GFP-gE(galK)R for

insertion of a C-terminal EGFP on gE (Table S3), where underlined sequences represent *galK* sequence homology.

Table S3. List of primers used for BAC recombineering.

Stage	Primer Name	Sequence (5'→3')
1	gEF-galK	CGTCTGTATACCCCCGTAGCGATGGGCATCAATCTCGCCGCCAGCTCACACCTGTTGACAATTAATCATCGGCA
	gER-galK	ACGGACGAATCGGAGGCCTGGGAGTAACGGCGATCGGGCCTTCCGGATCCTCAGCACTGTCCTGCTCCTT
	GFP-gE(galK)F	GAAGGCCCGATCGCCGTTACTCCCAGGCCTCCGATTCGTCCGTCTTCTGGCCTGTTGACAATTAATCATCGGCA
	GFP-gE(galK)R	CGGCGGTCGCCCAGTTCGGCGACCGACGTGGGGCCTCGGGATGGGGCGCCTCAGCACTGTCCTGCTCCTT
2	gE[AA]s	CGTCTGTATACCCCCGTAGCGATGGGCATCAATCTCGCCGCCAGCTCACAGCCGCTGGATCCGGAAGGCCCGATCGCCGTTACTCCCAGGCCTCCGATTCGTCCGT
	gE[AA]as	ACGGACGAATCGGAGGCCTGGGAGTAACGGCGATCGGGCCTTCCGGATCCAGCGGCTGTGAGCTGGCGGCGAGATTGATGCCCATCGCTACGGGGGTATACAGACG
	GFP-gE(BACTag)F	GAAGGCCCGATCGCCGTTACTCCCAGGCCTCCGATTCGTCCGTCTTCTGGTCGCCCTTCGGAGAAGGACAAGGACAAG
	GFP-gE(BACTag)R	CGGCGGTCGCCCAGTTCGGCGACCGACGTGGGGCCTCGGGATGGGGCGCCTTACTTGTACAGCTCGTCCATGCCGAG

Second stage recombination cassettes were generated in two ways. For HSV-1(17-37)-gE$_{TF/AA}$, the oligonucleotides gE[AA]s/gE[AA]as were annealed together and delivered to SW102 cells by electroporation as described (7) (Table S3). Underlined sequence indicates mutant nucleotides for the T530A/F531A mutations. For HSV-1(17-37)-gE-GFP and HSV-1(17-37)-gE$_{TF/AA}$-GFP, the primers GFP-gE(BACTag)F/GFP-gE(BACTag)R were used to amplify a linker and EGFP sequence from plasmid pEGFP-N1+gE(CT) (Table S3). Underlined sequence indicates homology to the linker/EGFP sequence encoded by pEGFP-N1+gE(CT). The Strain 17 U$_S$8 sequence was cloned between the EcoRI and KpnI sites of pEGFP-N1 (Clontech) with a flexible hydrophilic SPFGEGQGQGQGPGRGYAYRS linker (11) incorporated between the U$_S$8 and EGFP coding sequences. This PCR product was then delivered by electroporation to cells harboring the parental HSV-1(17-37) BAC or the HSV-1(17-37)-gE$_{TF/AA}$ BAC.

Bacterial clones that had successfully recombined to replace the *galK* sequence with the desired T530A/F531A mutation or linker/EGFP insertion were identified by restriction digestion fragmentation patterns using HindIII-HF, AseI or BstBI as well as PCR amplification of the U$_S$8-U$_S$9 open reading frames. PCR products of second-stage recombinants were sequence verified at the Australian Genome Research Facility (AGRF), Westmead, Sydney, Australia.

Parental HSV-1(17-37) BAC DNA and each recombinant BAC DNA construct were separately co-transfected into Vero cells with a Cre recombinase-expressing plasmid to release the viral genome from the BAC and generate infectious virus (10).

3. Recombinant Virus Characterization

3.1 Isolation and Screening of Viral DNA

For each virus, a T75 flask was seeded with Vero cells to be ~80% confluent at the time of infection. Cells were infected for 1 hour at 37°C (5% CO_2) at an MOI of 1 using 3 mL inoculum volumes made in Virus Growth Medium (DMEM + 2% (v/v) FBS + 100 U/mL penicillin + 100 µg/mL streptomycin). After 1 hour, flasks were topped up with an extra 9 mL of Virus Growth Medium and incubated at 37°C (5% CO_2) until CPE was extensive (~24 hours). Cells were

scraped into the medium and transferred to a 50 mL Falcon tube and sonicated twice for 60 seconds (80% output, 30 second rest on ice between pulses) using a Digital 450 Sonifier (Branson) fitted with a Cup Horn. Cell debris was pelleted by centrifugation at 1200 g for 5 minutes. 1 mL of a 25% (w/v) sucrose solution (in HBSS) was added to a 13.2 mL Beckman Centrifuge Ultra-Clear™ Tube (14 x 89 mm, #344059) before the ~12 mL virus supernatant was gently layered on top of this cushion. Viruses were ultracentrifuged at 210000 g (4°C, 1 hour) in a SW 41 Ti rotor (Beckman Coulter) with delayed deceleration before the cushion and supernatant were aspirated to isolate the virus pellet.

This pellet was resuspended in 1 mL NTE Buffer (0.5 mM NaCl, 10 mM Tris-HCl (pH 7.5), 5 mM EDTA (pH 8.0)) and lysed by the addition of SDS (final concentration 2.5% w/v) and EDTA (final concentration of 10 mM) before incubating for 5 minutes in a 37°C water bath. Viral DNA was separated from protein and lipid membranes by phenol-chloroform extraction, performed twice with phenol:chloroform:isoamyl alcohol (25:24:1) saturated with TE (pH 8.0). Resuspended viral pellets in NTE/SDS/EDTA were combined 1:1 with phenol solution and mixed by inversion for 20 seconds and centrifuged at 16000 g for 5 minutes. The upper DNA-containing aqueous layer was collected and phenol-chloroform-extracted once more in the same manner.

Viral DNA was then ethanol precipitated by the addition of 0.1 volumes of 3 M sodium acetate and 2 volumes of 100% ethanol. Samples were mixed and centrifuged at 21100 g (10 minutes) and the pellets washed with 70% ethanol. Samples were centrifuged once more before ethanol was aspirated and the pellets air dried. DNA was resuspended in 20 µL of sterile Milli-Q H_2O containing 50 µg/mL RNase A and stored at -20°C until required for further use.

DNA was assessed by restriction enzyme digestion, PCR screening and Sanger sequencing to ensure excision of the BAC backbone had occurred and intentional substitutions/insertions were maintained.

3.2 Single-Step Growth Kinetics

Single-step growth kinetics were performed as described previously (9). Experiments were performed as for multi-step growth kinetics but with inocula made up to an MOI of 5 and virus harvest timepoints at 4, 8, 12 and 24 hpi. Viral titers were determined by plaque assay.

3.3 Viral Protein Expression

Characterization of viral protein expression was performed as previously described (10). 6-well plates were seeded with Vero cells to reach confluency at 24 hours. Cells were infected for 1 hour (37°C, 5% CO_2) with virus at an MOI of 5 made up in Virus Growth Medium. Cells were washed once with sterile DPBS and overlaid with DMEM supplemented with 10% (v/v) FBS. At 24 hpi, medium was aspirated and cells washed once with DPBS. 100 µL of Virus Lysis Buffer (10 mM Tris/Cl pH 7.5, 150 mM NaCl, 0.5 mM EDTA, 0.5% NP-40 (IGEPAL® CA-630), 1X Mammalian Protease Inhibitor Cocktail (Sigma-Aldrich), 1X PhosSTOP (Roche)) was added to the cells which were then scraped into a pre-chilled Eppendorf tube. Tubes were incubated on ice with extensive pipetting every 10 minutes for a total 30 minutes before cell debris was pelleted by centrifugation at 20000 g (4°C, 10 minutes). Protein-containing supernatants were combined with an equal volume of SDS Sample Buffer (2X) and boiled at 95°C for 10 minutes before SDS-PAGE analysis.

Supplementary Methods References

1. UniProt C. UniProt: a worldwide hub of protein knowledge. Nucleic Acids Res. 2019;47(D1):D506-D15, DOI: 10.1093/nar/gky1049.
2. Chen B, Brinkmann K, Chen Z, Pak CW, Liao Y, Shi S, et al. The WAVE regulatory complex links diverse receptors to the actin cytoskeleton. Cell. 2014;156(1-2):195-207, DOI: 10.1016/j.cell.2013.11.048.
3. Buchan DWA, Jones DT. The PSIPRED Protein Analysis Workbench: 20 years on. Nucleic Acids Res. 2019;47(W1):W402-W7, DOI: 10.1093/nar/gkz297.
4. Jones DT. Protein secondary structure prediction based on position-specific scoring matrices. J Mol Biol. 1999;292(2):195-202, DOI: 10.1006/jmbi.1999.3091.
5. Jones DT, Cozzetto D. DISOPRED3: precise disordered region predictions with annotated protein-binding activity. Bioinformatics. 2015;31(6):857-63, DOI: 10.1093/bioinformatics/btu744.
6. Gierasch WW, Zimmerman DL, Ward SL, Vanheyningen TK, Romine JD, Leib DA. Construction and characterization of bacterial artificial chromosomes containing HSV-1 strains 17 and KOS. J Virol Methods. 2006;135(2):197-206, DOI: 10.1016/j.jviromet.2006.03.014.
7. Warming S, Costantino N, Court DL, Jenkins NA, Copeland NG. Simple and highly efficient BAC recombineering using galK selection. Nucleic Acids Res. 2005;33(4):e36, DOI: 10.1093/nar/gni035.
8. de Oliveira AP, Glauser DL, Laimbacher AS, Strasser R, Schraner EM, Wild P, et al. Live visualization of herpes simplex virus type 1 compartment dynamics. J Virol. 2008;82(10):4974-90, DOI: 10.1128/JVI.02431-07.
9. Kelly BJ, Bauerfeind R, Binz A, Sodeik B, Laimbacher AS, Fraefel C, et al. The interaction of the HSV-1 tegument proteins pUL36 and pUL37 is essential for secondary envelopment during viral egress. Virology. 2014;454-455:67-77, DOI: 10.1016/j.virol.2014.02.003.
10. Diefenbach RJ, Davis A, Miranda-Saksena M, Fernandez MA, Kelly BJ, Jones CA, et al. The Basic Domain of Herpes Simplex Virus 1 pUS9 Recruits Kinesin-1 To Facilitate Egress from Neurons. J Virol. 2016;90(4):2102-11, DOI: 10.1128/JVI.03041-15.
11. Taylor MP, Kramer T, Lyman MG, Kratchmarov R, Enquist LW. Visualization of an alphaherpesvirus membrane protein that is essential for anterograde axonal spread of infection in neurons. mBio. 2012;3(2), DOI: 10.1128/mBio.00063-12.

Supplementary Data

Table S1. HSV-1 membrane protein WIRS motif consensus analysis.

Protein[1]	Gene	HSV-1 Strain 17 Length (aa)	Cytoplasmic Region(s) (aa)	WIRS Residues (aa)
glycoprotein B	U$_L$27	904	796-904	None
glycoprotein C	U$_L$44	511	498-511	None
glycoprotein D	U$_S$6	394	362-394	None
glycoprotein E	U$_S$8	550	441-550	LTTF (aa528-531)
glycoprotein G	U$_S$4	238	211-238	None
glycoprotein H	U$_L$22	838	825-838	None
glycoprotein I	U$_S$7	390	298-390	None
glycoprotein J	U$_S$5	92	71-92	None
glycoprotein K	U$_L$53	338	141-212, 265-301	None
glycoprotein L[2]	U$_L$1	224	N/A	None
glycoprotein M	U$_L$10	473	1-32, 112-137, 185-216, 272-280, 340-473	None
glycoprotein N	U$_L$49.5	91	77-91	None
lipid anchor protein pUL11[3]	U$_L$11	96	N/A	None
envelope protein pU$_L$20	U$_L$20	222	1-63, 119-130, 201-222	None
transmembrane protein pU$_L$43[4]	U$_L$43	417	N/A	None
envelope protein pU$_L$45[5]	U$_L$45	172	1-27	None
lipid anchor protein pU$_L$51[3]	U$_L$51	244	N/A	None
transmembrane protein pU$_L$56[4]	U$_L$56	234	N/A	None
envelope protein pU$_S$9[5]	U$_S$9	90	1-67	None

[1] Protein sequences of the envelope proteins of HSV-1 Strain 17 were downloaded from UniProt and were assessed for the presence of WIRS motif consensus sequences in their cytoplasmic regions only. Sequences were obtained from the following UniProt Knowledgebase entries: gB, P10211; gC, P10228; gD, Q69091; gE, P04488; gG, P06484; gH, P06477; gI, P06487; gJ, P06480; gK, P68331; gL, P10185; gM, P04288; gN, O09800; lipid anchor protein pU$_L$11, P04289; envelope protein pU$_L$20, P10204; transmembrane protein pU$_L$43, P10227; envelope protein pU$_L$45, P10229; lipid anchor protein pU$_L$51, P10235; transmembrane protein pU$_L$56, P10240; envelope protein pU$_S$9, P06481. aa = amino acid.

[2] Glycoprotein L is a membrane-associated protein that heterodimerises with glycoprotein H to facilitate fusion of the viral and plasma membranes.

[3] These proteins are anchored to the intraviral side of the viral envelope.

[4] These proteins include transmembrane domains but do not form part of the viral envelope.

[5] These proteins are type II membrane proteins and so their cytoplasmic regions are at the N-terminus of the protein.

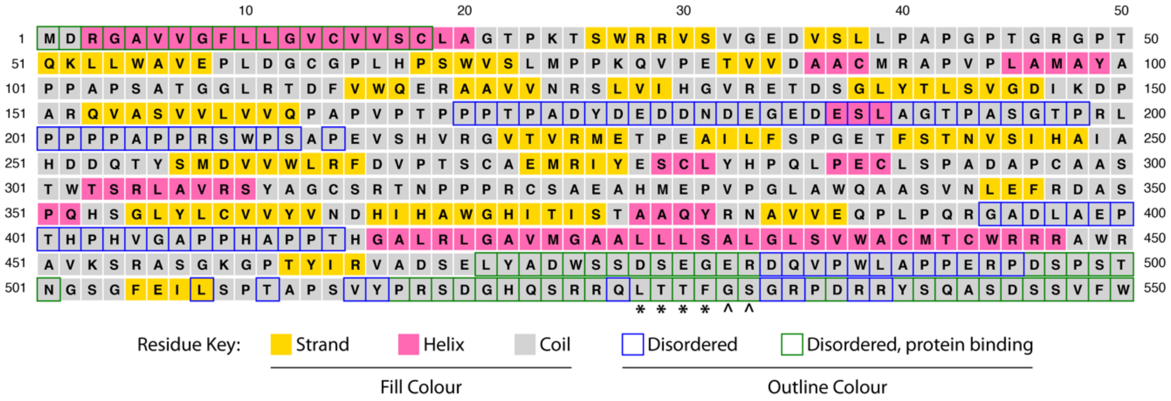

Figure S1. Secondary structure and disordered region prediction of the HSV-1 gE protein sequence. The HSV-1 Strain 17 gE protein sequence was uploaded to the PSIPRED Workbench server and subjected to PSIPRED 4.0 and DISOPRED3 analyses using default parameter settings. '*' symbols indicate key WIRS motif residues and '^' represents the X-X flanking residues of the gE(CT) WIRS. The L-T-T-F sequence at amino acids 528-531 of gE was predicted to be a coiled (but disordered) protein binding sequence flanked by disordered Q and G-R residues, conforming to the published consensus.

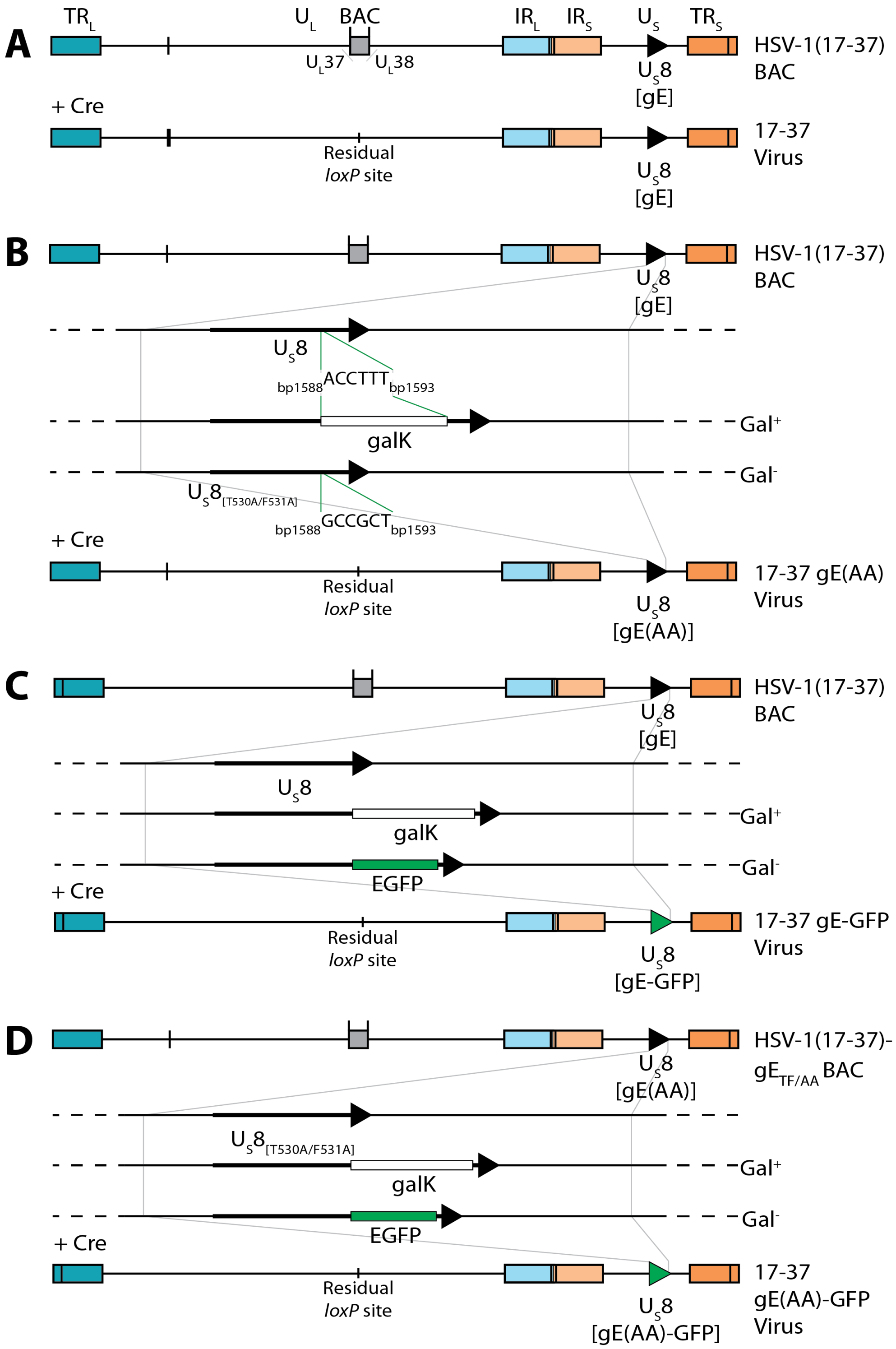

Figure S2. Schematic representation of recombinant BAC-derived HSV-1. (**A**) Two-stage galactokinase selection/counter-selection was used to engineer mutations/insertions of interest in the parental HSV-1 17-37(BAC). (**B**) A *galK* targeting cassette was inserted into the T530/F531A coding sequence of gE by homologous recombination followed by selection for galactokinase-positive (Gal^+) recombinants. This sequence was then replaced by mutant T530A/F531A coding sequence by homologous recombination and subsequent counterselection for galactokinase-negative (Gal^-) recombinants. BAC sequences were eliminated by Cre/*loxP* cleavage when introduced into Vero cells. (**C**, **D**) HSV-1(17-37) and HSV-1(17-37)-$gE_{TF/AA}$ BAC DNA were used to generate GFP-tagged wild-type or WIRS-mutant gE viruses, respectively. Within each panel the first row describes the starting BAC DNA and the final row describes the final virus labelled with its simplified name, having lost the BAC backbone by Cre-lox recombination within the mammalian cell.

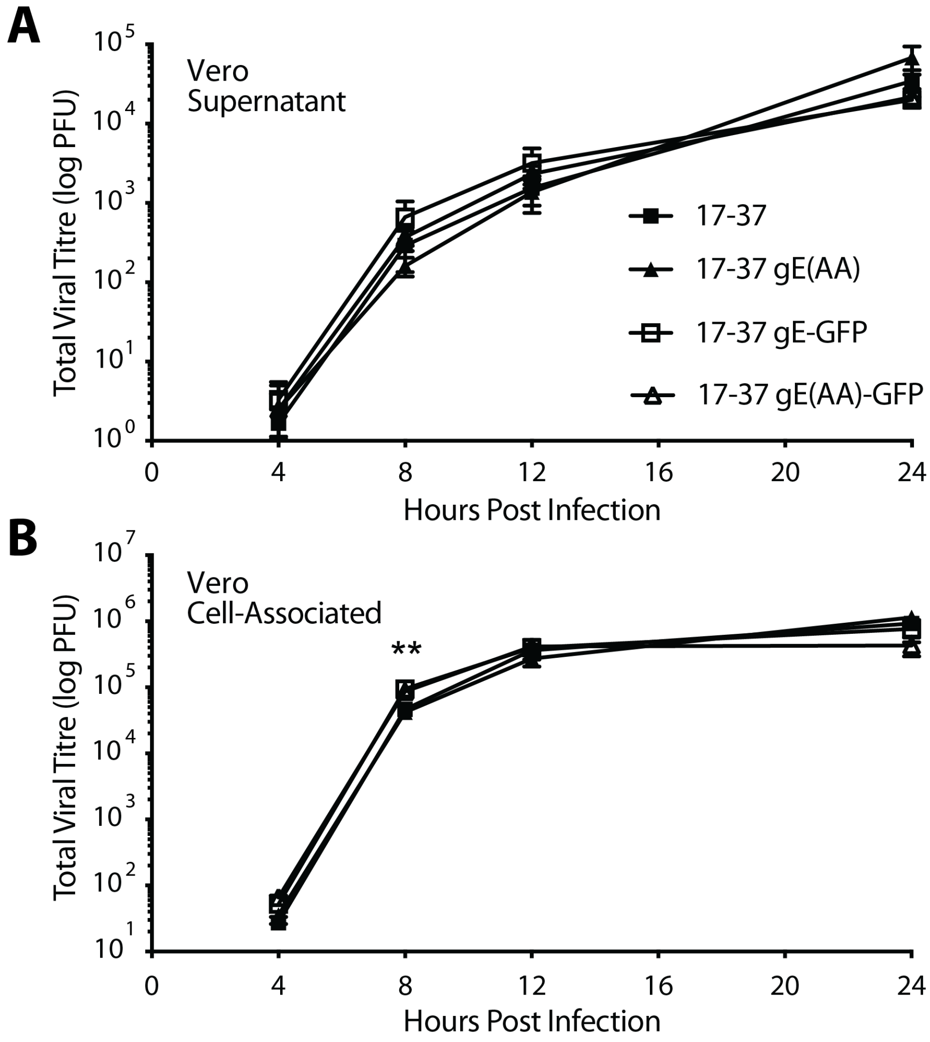

Figure S3. Recombinant viruses demonstrate wild-type growth kinetics. Confluent monolayers of Vero cells were infected at an MOI of 5 with parental 17-37 or the indicated recombinant viruses for 1 hour. Cells were incubated with Virus Inactivation Buffer for 2 minutes and wells washed twice with PBS. Cells were overlaid with 2 mL Virus Growth Kinetics Medium and incubated until harvest. Supernatant virus and cell-associated virus titers were determined separately and so at the times indicated, the supernatant was collected and frozen at -80°C and the cells washed twice with PBS before being scraped into 1 mL fresh medium and frozen at -80°C. Once all samples had been harvested, samples were sonicated and subsequently titred by plaque assay on Vero cells. Supernatant virus titers are presented in **A** and cell-associated titers in **B**. The legend in **A** is applicable to both figure panels. Error bars represent mean ± SEM (n = 3). In some cases, error bars are not visible since they are smaller than the size of the representative symbol. Two-tailed unpaired parametric t-tests were performed between each recombinant virus and its parental virus, $^{**}p < 0.01$.

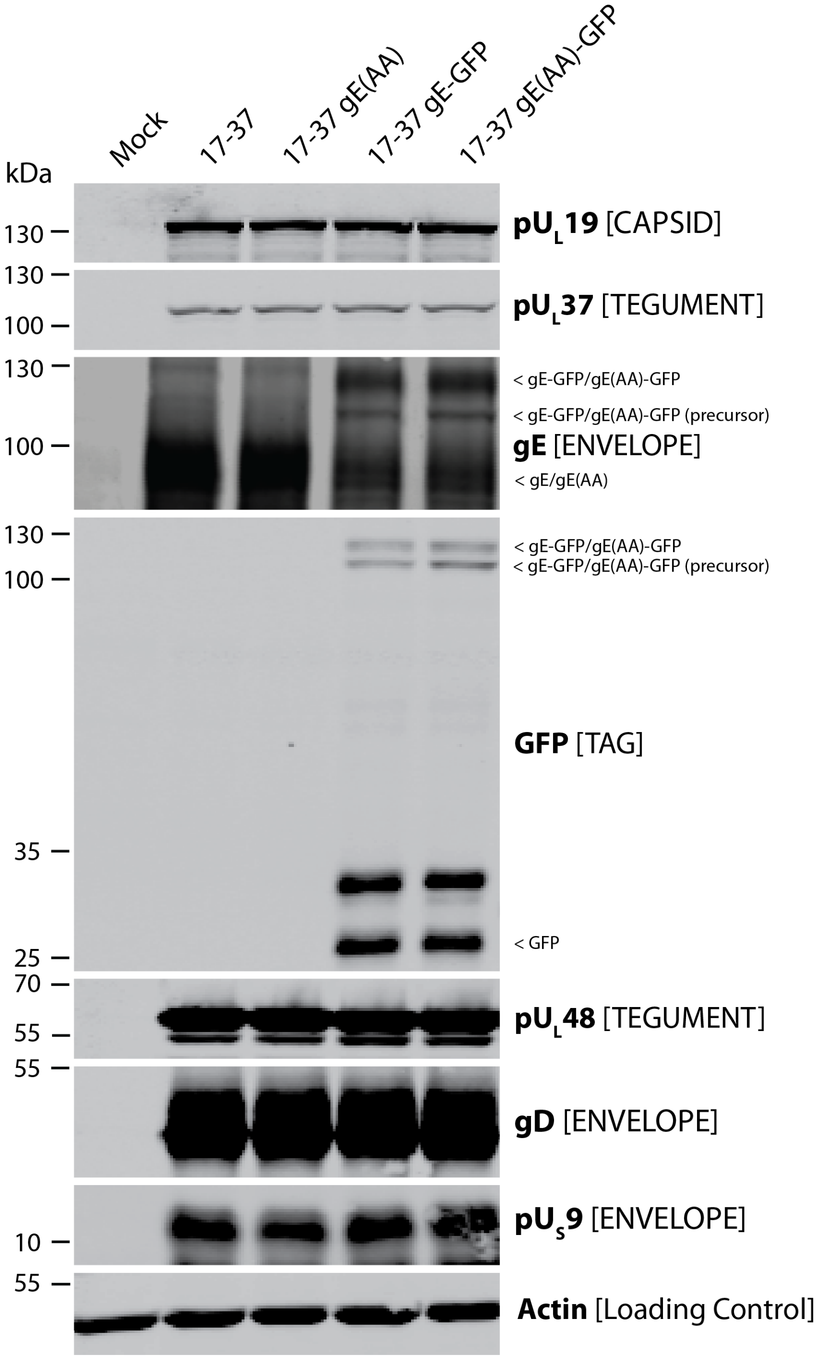

Figure S4. Immunoblot characterization of recombinant 17-37 HSV-1 protein production. Vero cells were infected for 24 hours at an MOI of 5. Cells were lysed and the soluble protein-containing supernatant used for immunoblot analysis with the indicated antibodies. Representative proteins of the three structural components of the HSV-1 virion (capsid, tegument and envelope) were assessed for any changes in protein expression levels caused by mutation of the gE coding sequence. For original uncropped gel images see additional supplementary file.

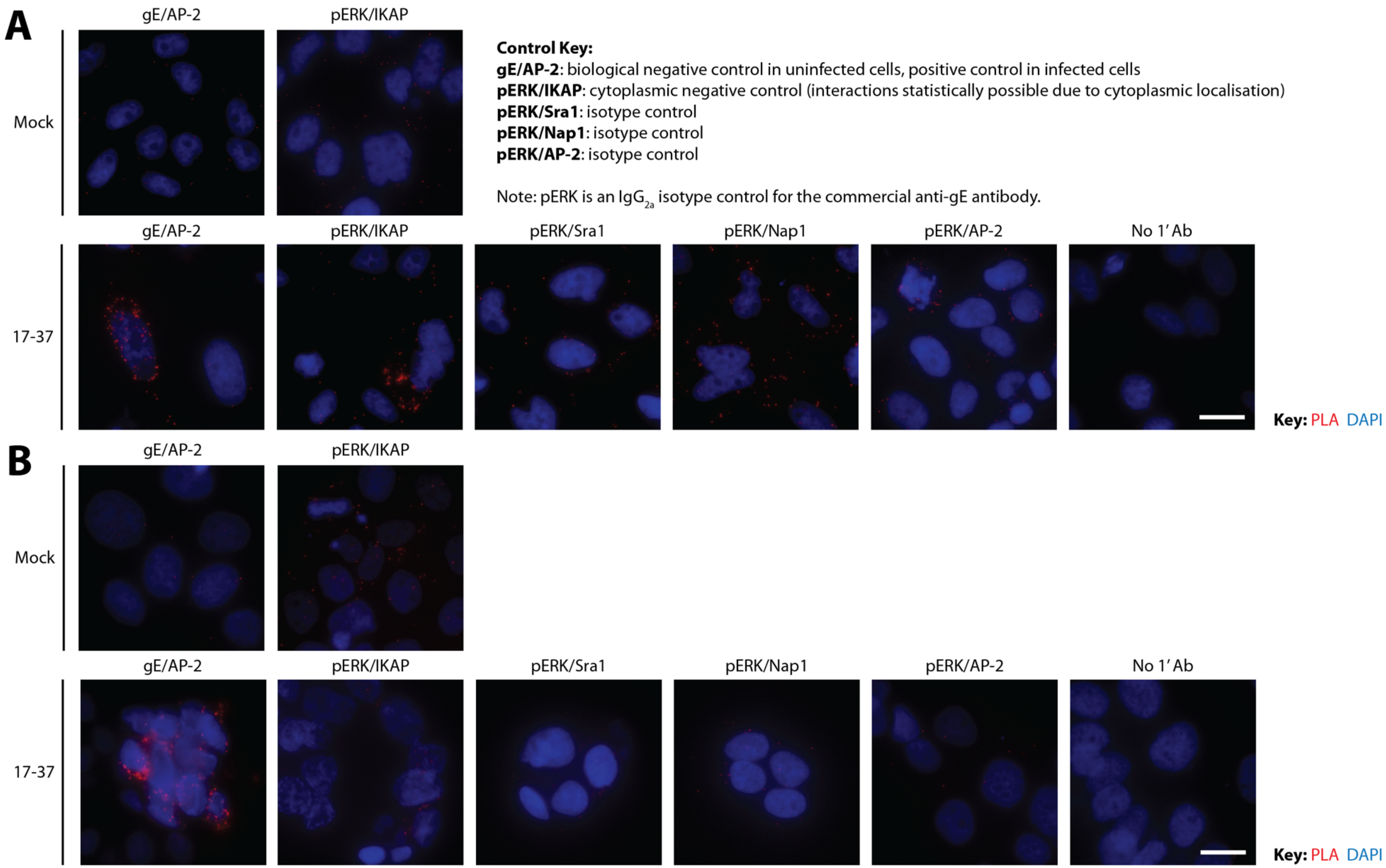

Figure S5. Proximity ligation analysis controls demonstrate low-level background of colocalizing signals in HeLa and HaCaT cells. HeLa (**A**) and HaCaT (**B**) cells were infected at an MOI of 2 or 1, respectively, and fixed at 24 hpi. Cells were processed using the Duolink® In Situ – Fluorescence Kit (Red, Mouse and Rabbit). Primary antibodies (rabbit and mouse pairs) were incubated with the cells as indicated before detection was performed using the kit. Distinct red puncta are representative of colocalisation events. Scale bars represent 20 µm. Images were captured on an Olympus BX53 Upright Microscope fitted with fluorescence filters.

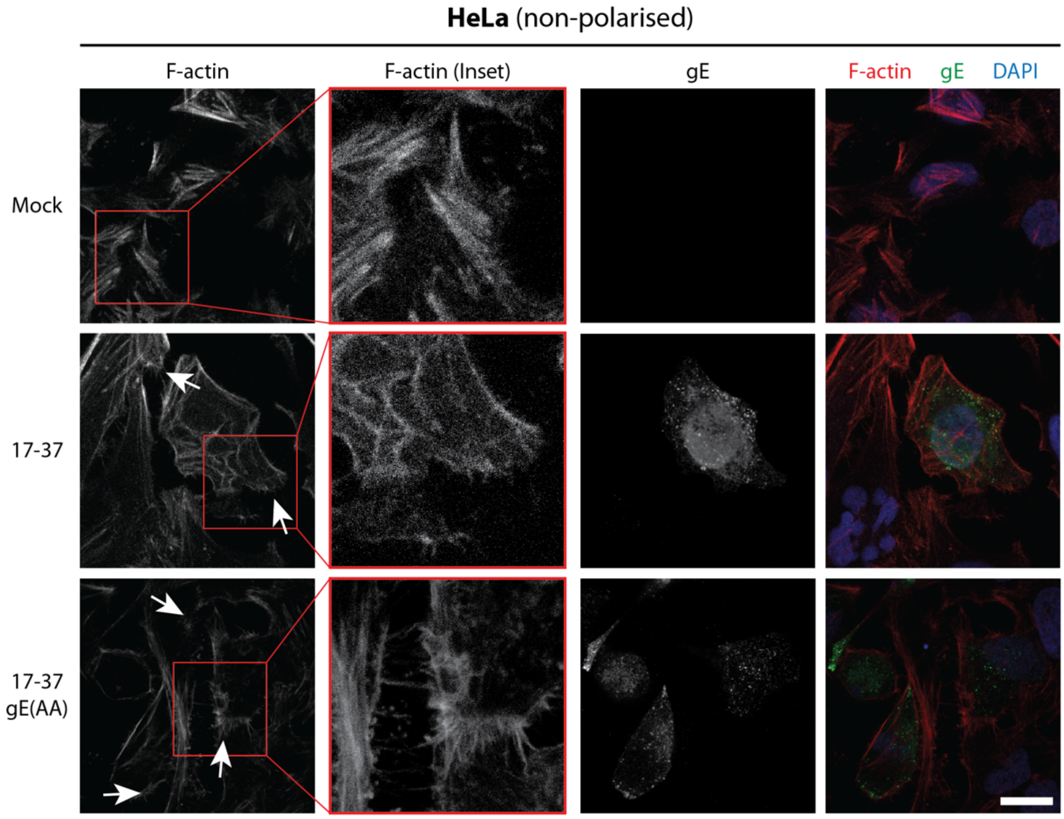

Figure S6. F-actin distribution is unchanged with WIRS-mutant gE. HeLa cells were synchronously infected with parental 17-37 or recombinant 17-37 gE(AA) viruses at an MOI of 3 for 24 hours before fixation. Cells were probed for viral gE and counterstained with phalloidin-AF633 (to stain F-actin; pseudocolored as red) and DAPI. Micrographs were captured on a Leica TCS SP5 II laser scanning confocal microscope. Scale bar represents 20 μm. Images are representative of at least 2 independent biological replicates. Arrows indicate cortical actin structures. Arrows point to filopodia formed after infection.

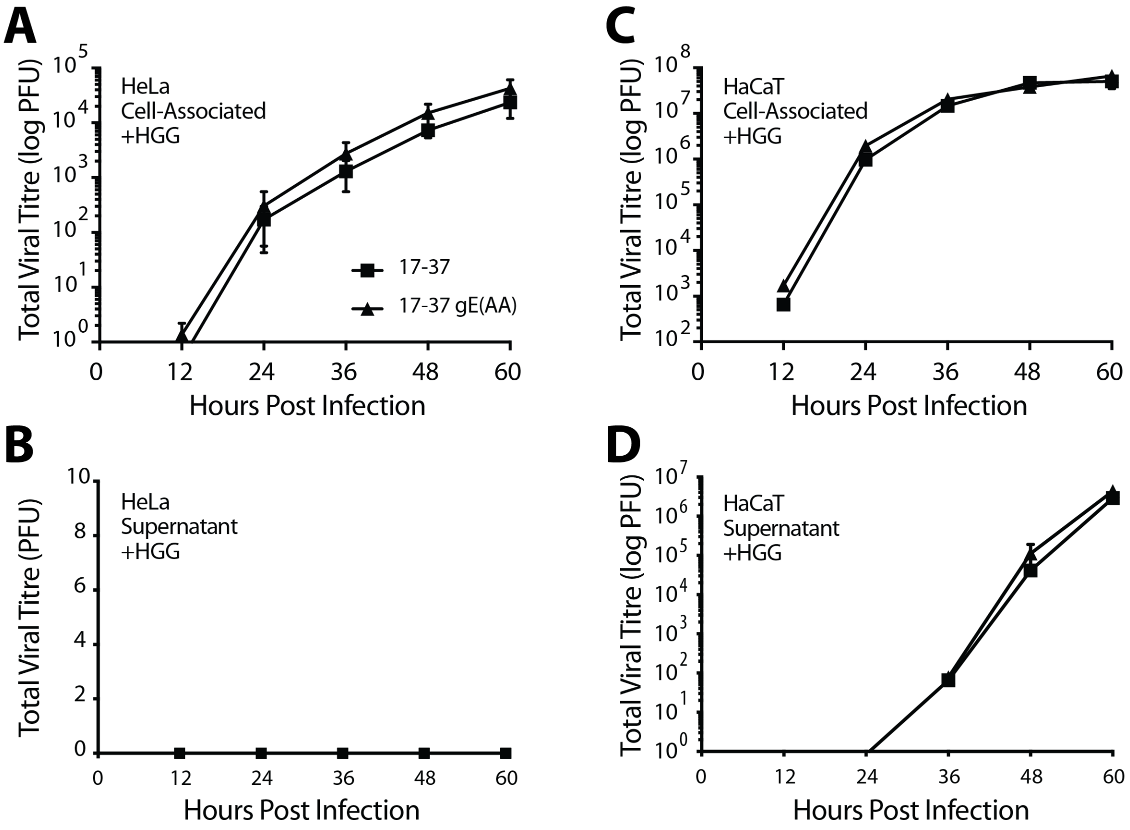

Figure S7. HGG neutralization of released virus in HeLa and HaCaT cells at 500 µg/mL. Confluent monolayers of HeLa (**A, B**) or HaCaT (**C, D**) cells were infected at an MOI of 0.001 with parental 17-37 (■) or 17-37 gE(AA) (▲) viruses for 1 hour. Cells were incubated with Virus Inactivation Buffer for 2 minutes and wells washed twice with PBS. Cells were overlaid with 2 mL Virus Growth Kinetics Medium supplemented with 500 µg/mL HGG and incubated until harvest. At the times indicated, media was collected and frozen at -80°C and the cells washed twice with PBS before being scraped into 1 mL fresh medium and frozen at -80°C. Once all samples had been harvested, samples were sonicated and subsequently titred by plaque assay on Vero cells. Error bars represent mean ± SEM (*n* = 3). Two-tailed unpaired parametric *t*-tests with Welch's correction were performed at each time point.

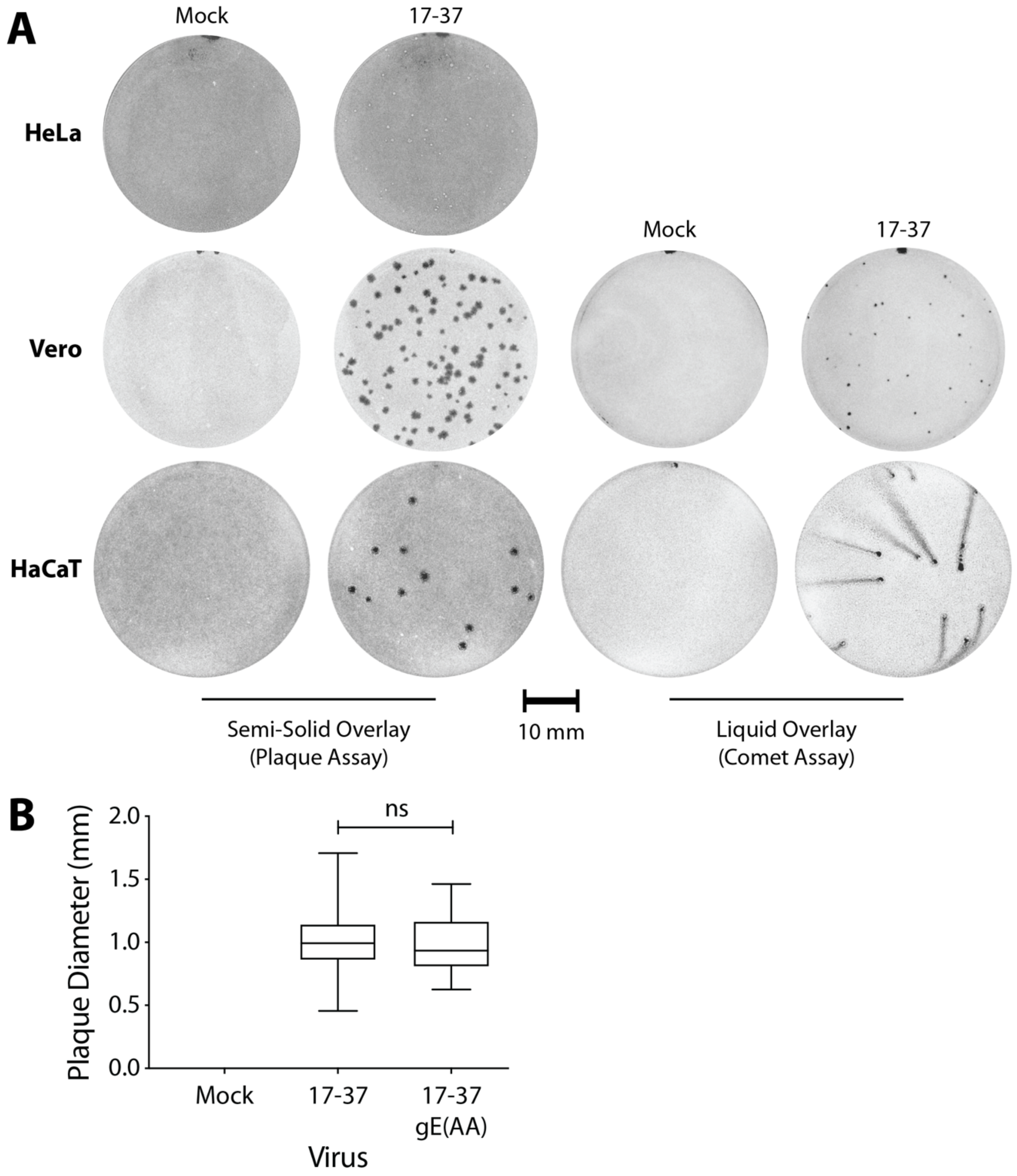

Figure S8. HSV-1 plaque and comet phenotypes in various cell types. In 6-well plates, HeLa, HaCaT or Vero cell monolayers were infected with 17-37 at ~100 PFU/well (for HeLa and Vero plaque assays), ~25 PFU/well (for Vero comet assays) or ~10 PFU/well (for HaCaT plaque and comet assays) for 2 hours before cells were overlaid with either a semi-solid carboxymethylcellulose overlay (plaque assay) or a liquid overlay (comet assay). At 72 hpi, overlays were removed, the cells were fixed in methanol and plaques/comets were visualized by crystal violet staining of the cell monolayer. Images were obtained on a ChemiDoc Touch (Bio-Rad). (**A**) Infected HeLa cells do not readily form measurable plaques at 72 hpi and were therefore not tested further for subsequent comet assays. Infected Vero and HaCaT cells produce measurable plaques by 72 hpi, but only HaCaT cells are amenable to comet formation in liquid overlays. (**B**) Plaque diameter in Vero cells was measured in FIJI. Data was statistically analyzed for n = 50 plaques using a two-tailed unpaired parametric *t*-test with Welch's correction (ns, non-significant).

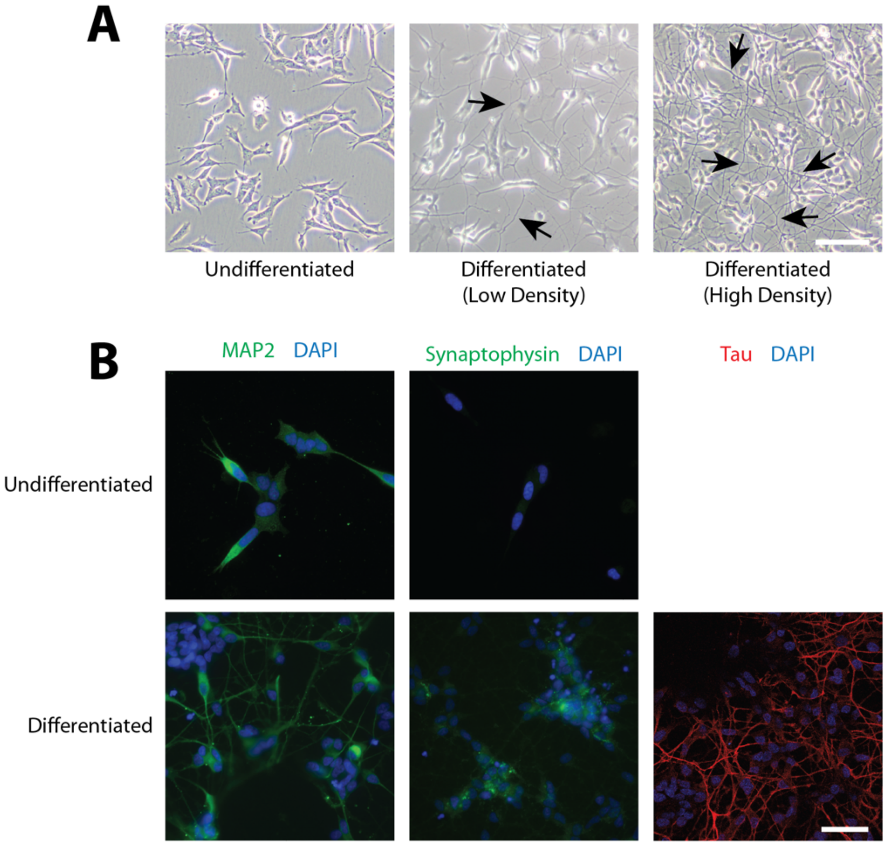

Figure S9. Morphological appearance of differentiated SH-SY5Y cells and expression of markers of cell maturity. (**A**) Undifferentiated SH-SY5Y cells were seeded onto PDL- and laminin-coated wells and overlaid with SH-SY5Y Differentiation Medium. Cells were incubated at 37°C (5% CO_2) for 6 days with medium refreshments every 48 hours. Images were collected on an Olympus CKX41 microscope after 6 days of differentiation at both a low seeding density (for microscopic analyses) or high seeding density (for protein-based assays) or 1 day of growth for undifferentiated samples. Arrows indicate presence of cytoplasmic extensions (neurites). Scale bar represents 100 µm. (**B**) Undifferentiated SH-SY5Y cells on glass coverslips (at 24 hours post-seeding) or differentiated SH-SY5Y cells grown on PDL- and laminin-coated glass coverslips (6 days post-seeding and differentiation) were fixed in IF Fixing Buffer and processed for immunofluorescence microscopy. Imaged on an Olympus BX53 Upright Microscope fitted with fluorescent filters, differentiated cells show redistributed MAP2 expression (from cytoplasm of undifferentiated cells to neurites following differentiation) and increased synaptophysin expression. Tau expression wasn't tested in undifferentiated cells due to antibody access during experimentation. Images collected on a Leica TCS SP5 II laser scanning confocal microscope show tau expression along neurites of differentiated cells as expected for mature SH-SY5Y neurons. Scale bar represents 50 µm.

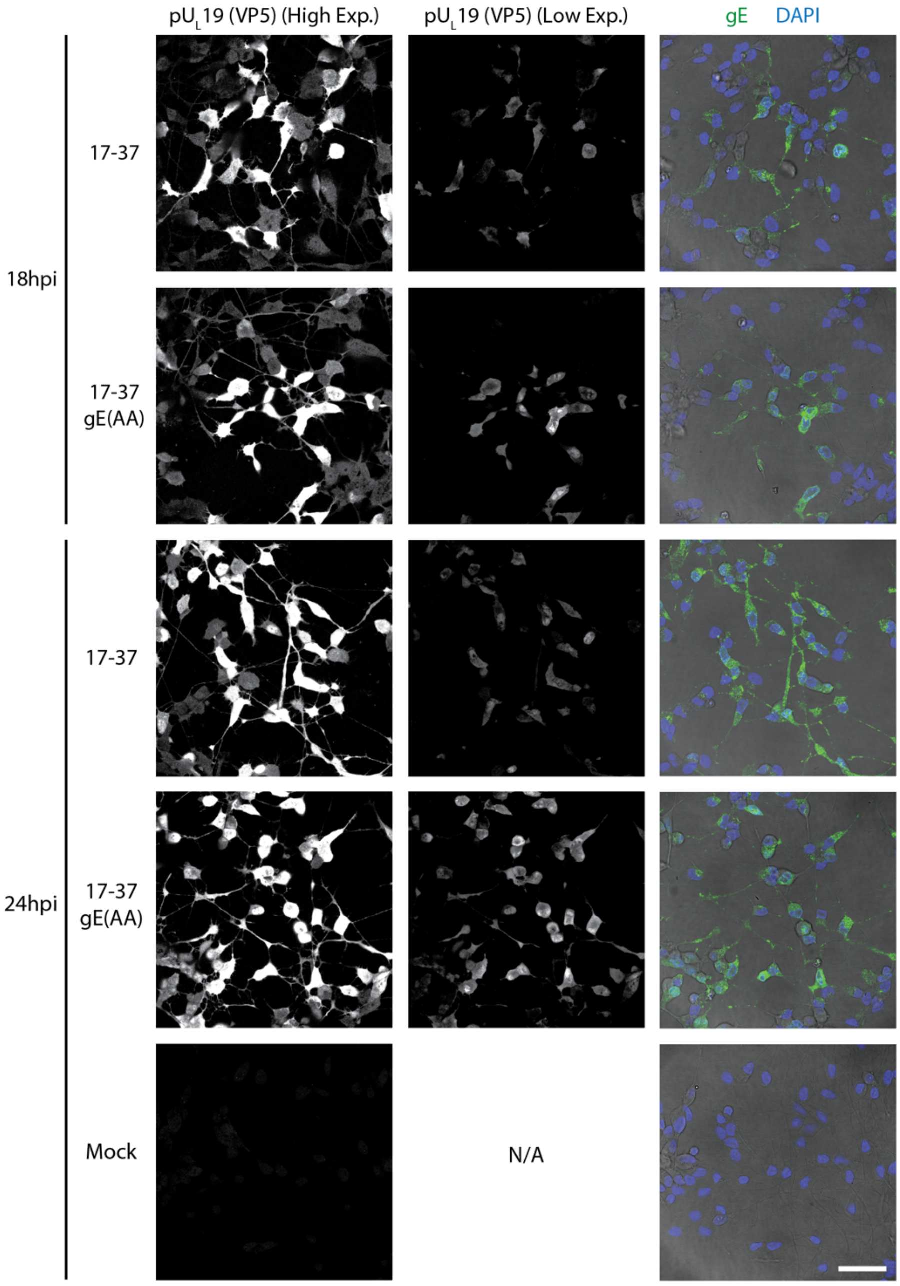

Figure S10. Supplementary panels for Figure 9 regarding gE/gE(AA) transport along neurites of dSH-SY5Y cells. Infected cells, as defined by successful co-staining of pU$_L$19 (VP5) and gE within the same field of view, were compared for their gE distribution: cell body only or both axon and cell body. Due to strong expression of the major capsid protein within the cell body of these cells, high and low exposure of the pU$_L$19 antibody channel was used to observe successful transport of viral capsid along neurites. The merged panels on the right are presented exactly as in Figure 9. Scale bar represents 50 µm.

Original Uncropped Gel Images

For Figure 3

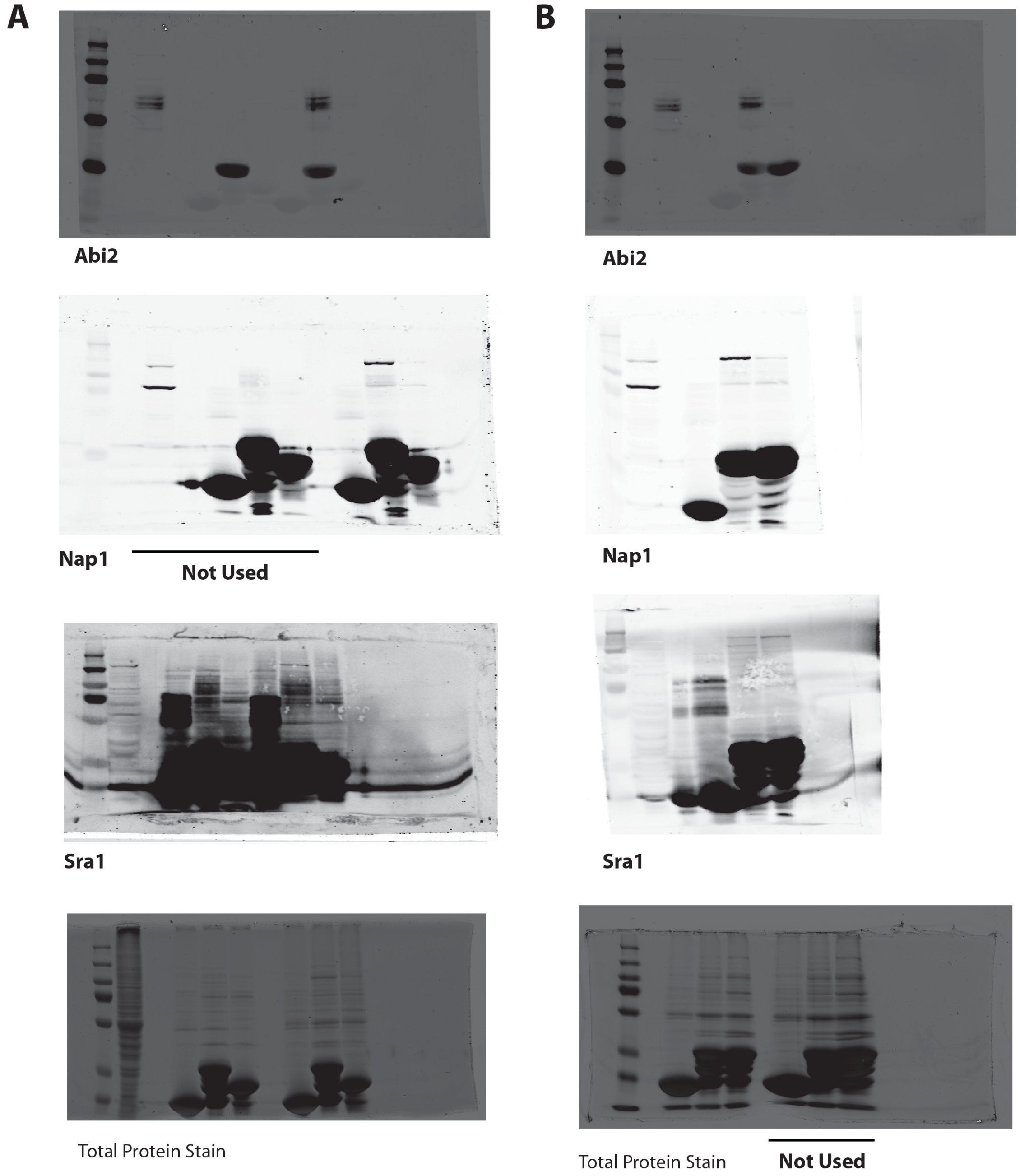

For Figure 4

Note: All blots have an empty well immediately following the ladder, but the pU$_S$9 HeLa blot has two empty wells after the ladder before sample loading begins.

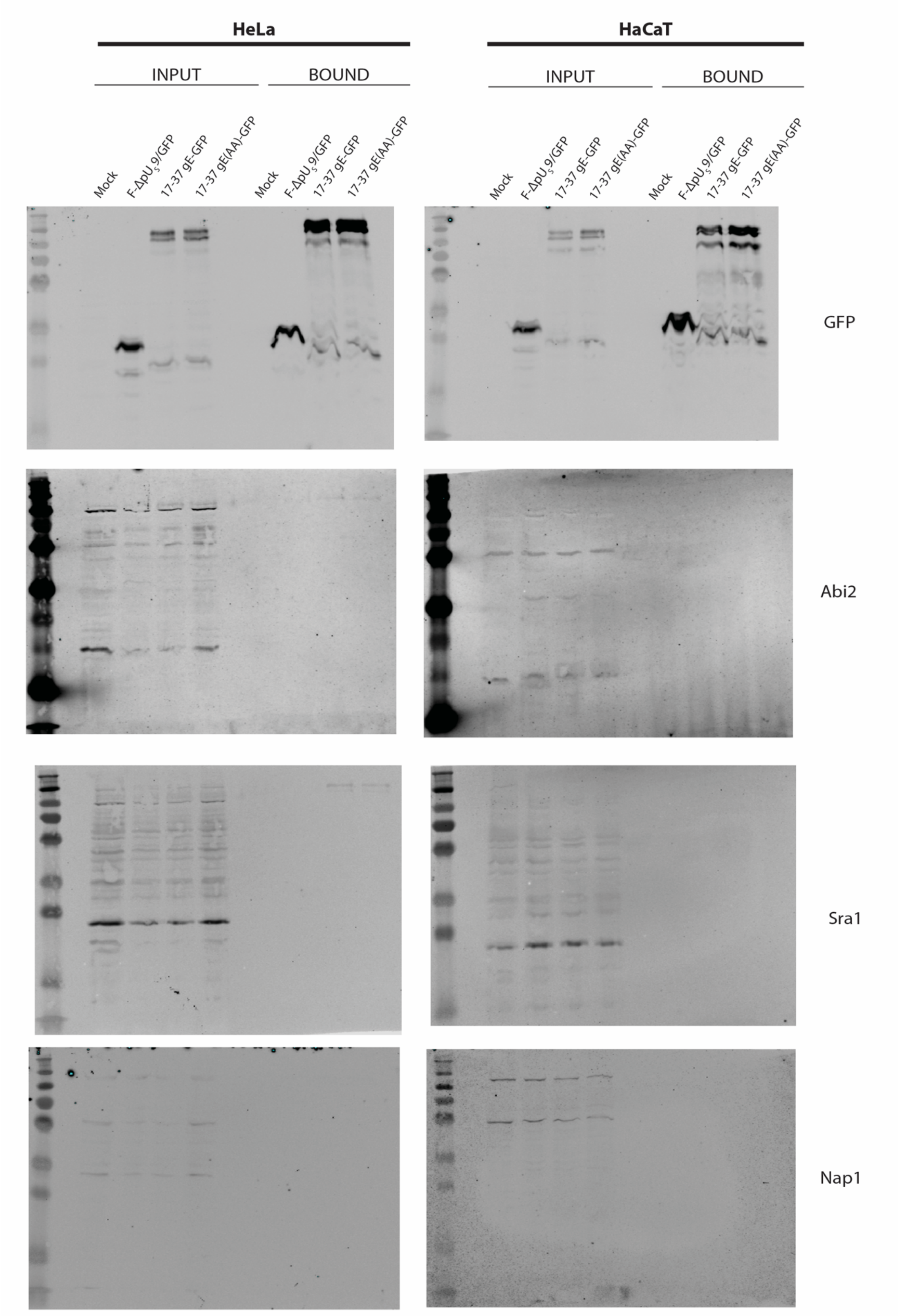

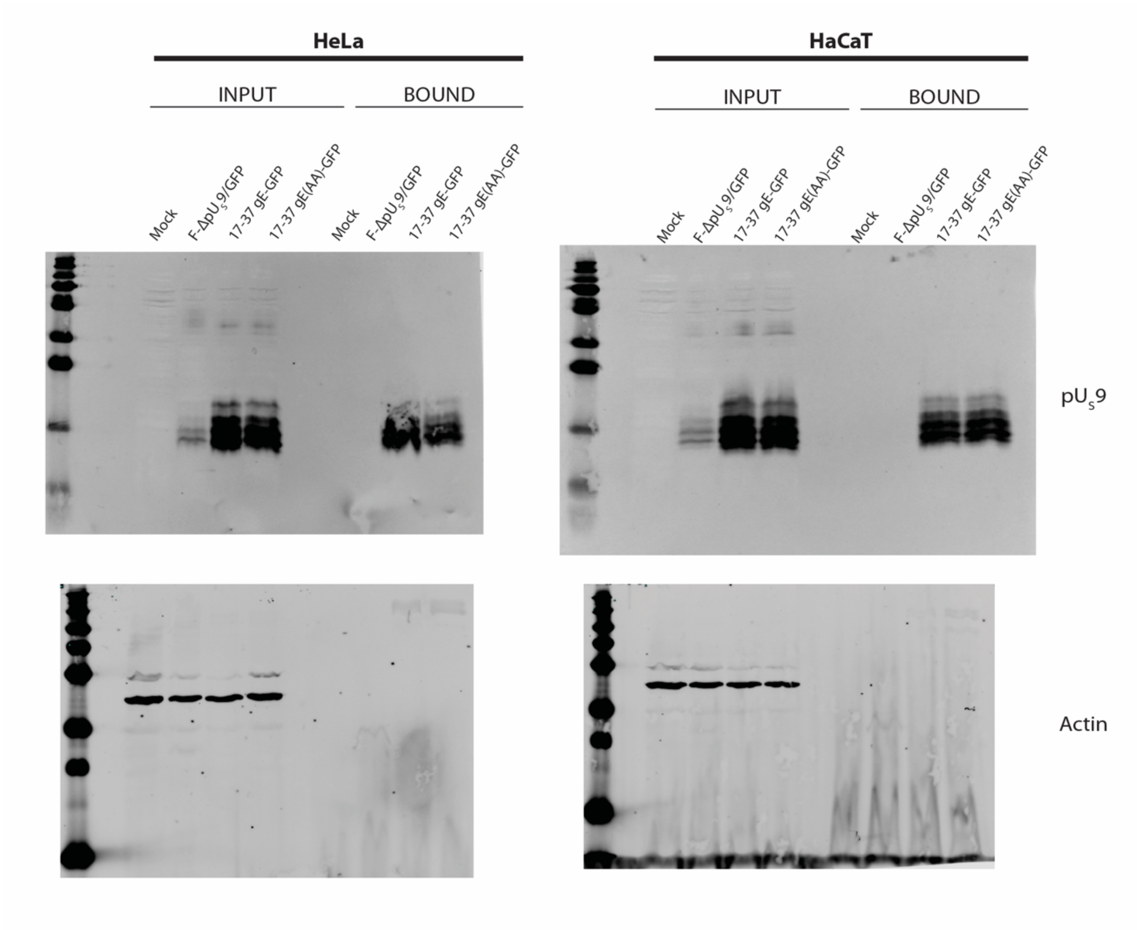
HeLa
HaCaT
INPUT
BOUND
INPUT
BOUND
Mock
F-ΔpUS9/GFP
17-37 gE-GFP
17-37 gE(AA)-GFP
Mock
F-ΔpUS9/GFP
17-37 gE-GFP
17-37 gE(AA)-GFP
pUS9
Actin

For Figure S4

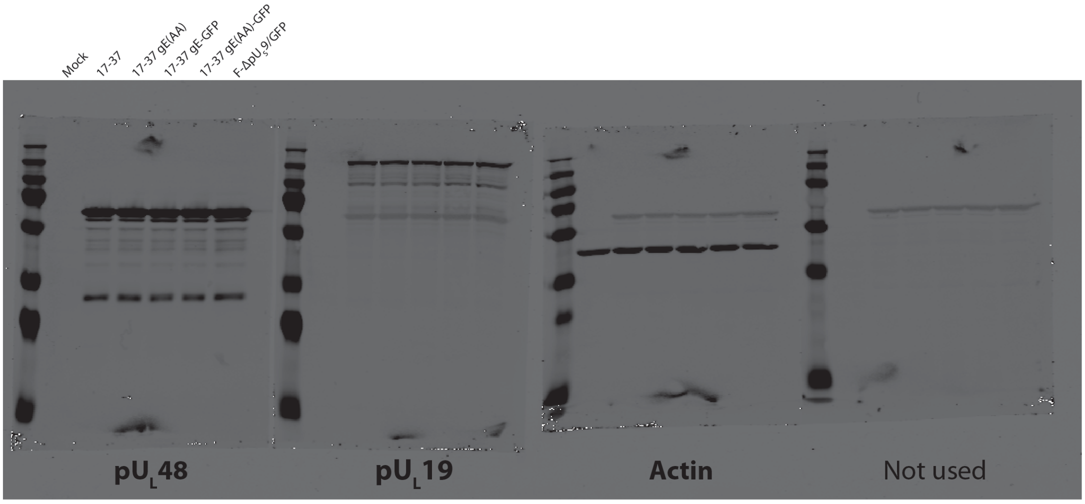

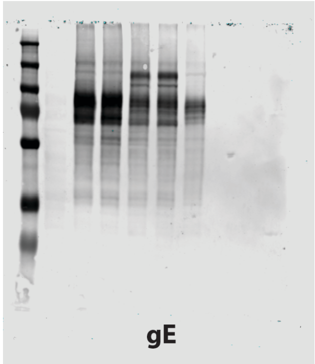

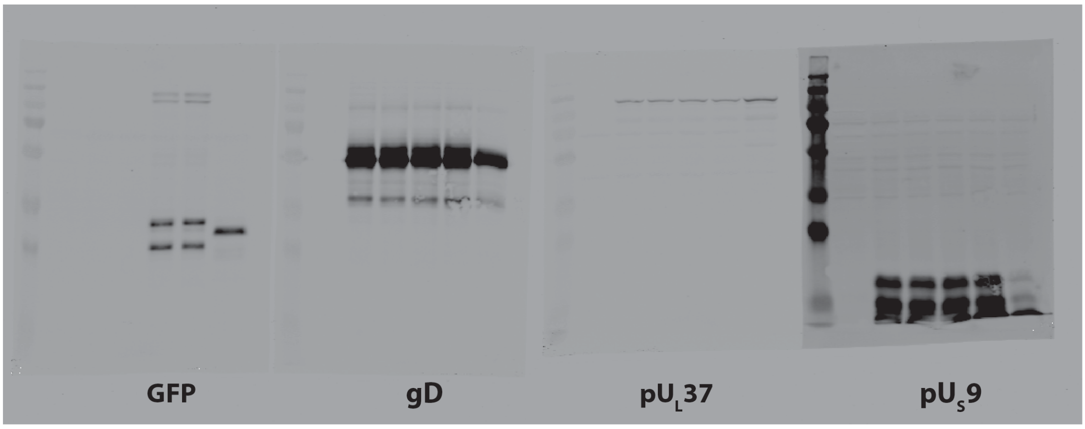

Supplement: Supplementary material 1 [file acmi-3-0206-s001.pdf]
